# Genome-wide annotation of the soybean WRKY family and functional characterization of genes involved in response to *Phakopsora pachyrhizi* infection

**DOI:** 10.1186/s12870-014-0236-0

**Published:** 2014-09-10

**Authors:** Marta Bencke-Malato, Caroline Cabreira, Beatriz Wiebke-Strohm, Lauro Bücker-Neto, Estefania Mancini, Marina B Osorio, Milena S Homrich, Andreia Carina Turchetto-Zolet, Mayra CCG De Carvalho, Renata Stolf, Ricardo LM Weber, Gastón Westergaard, Atílio P Castagnaro, Ricardo V Abdelnoor, Francismar C Marcelino-Guimarães, Márcia Margis-Pinheiro, Maria Helena Bodanese-Zanettini

**Affiliations:** Programa de Pós-Graduação em Genética e Biologia Molecular, Universidade Federal do Rio Grande do Sul (UFRGS), Porto Alegre, Brazil; Instituto de Agrobiotecnologia Rosario SA, Rosario, Argentina; Empresa Brasileira de Pesquisa Agropecuária (Embrapa Soja), Londrina, Brazil; Estación Experimental Agroindustrial Obispo Colombres (EEAOC), Tucumán, Argentina

**Keywords:** *Glycine max*, Genetic transformation, Fungus resistance, Transcription factors, Asian Soybean Rust, Functional analysis

## Abstract

**Background:**

Many previous studies have shown that soybean WRKY transcription factors are involved in the plant response to biotic and abiotic stresses. *Phakopsora pachyrhizi* is the causal agent of Asian Soybean Rust, one of the most important soybean diseases. There are evidences that WRKYs are involved in the resistance of some soybean genotypes against that fungus. The number of *WRKY* genes already annotated in soybean genome was underrepresented. In the present study, a genome-wide annotation of the soybean WRKY family was carried out and members involved in the response to *P. pachyrhizi* were identified.

**Results:**

As a result of a soybean genomic databases search, 182 *WRKY*-encoding genes were annotated and 33 putative pseudogenes identified. Genes involved in the response to *P. pachyrhizi* infection were identified *using* superSAGE, RNA-Seq of microdissected lesions and microarray experiments. Seventy-five genes were differentially expressed during fungal infection. The expression of eight *WRKY* genes was validated by RT-qPCR. The expression of these genes in a resistant genotype was earlier and**/**or stronger compared with a susceptible genotype in response to *P. pachyrhizi* infection. Soybean somatic embryos were transformed in order to overexpress or silence *WRKY* genes. Embryos overexpressing a *WRKY* gene were obtained, but they were unable to convert into plants. When infected with *P. pachyrhizi*, the leaves of the silenced transgenic line showed a higher number of lesions than the wild-type plants*.*

**Conclusions:**

The present study reports a genome-wide annotation of soybean WRKY family. The participation of some members in response to *P. pachyrhizi* infection was demonstrated. The results contribute to the elucidation of gene function and suggest the manipulation of WRKYs as a strategy to increase fungal resistance in soybean plants.

**Electronic supplementary material:**

The online version of this article (doi:10.1186/s12870-014-0236-0) contains supplementary material, which is available to authorized users.

## Background

Soybean (*Glycine max*) is one of the most important crops in the world. At present, one of the major diseases affecting soybean production is Asian Soybean Rust (ASR), which results from infection with *Phakopsora pachyrhizi* [1]. Under conditions that are favorable for fungal propagation, infection results in yield losses ranging from 10 to 80% [2-4].

Three infection types have been described on soybean accessions inoculated with *P. pachyrhizi*: (1) susceptible reaction characterized by “tan” lesions with many uredinia and prolific sporulation; (2) resistant reaction typified by reddish brown lesions with few uredinia and little to moderate sporulation; and (3) resistant reaction with no visible lesions or uredinia, conferring the immune phenotype [5,3]. Six single dominant genes (*Rpp1* to *Rpp6*) conditioning soybean resistance and/or immunity to *P. pachyrhizi* have been identified so far [5-14]. The effectiveness of these genes is limited through virulent ASR isolates that are able to overcome the resistance mechanism conferred by each of them [1,15]. For this reason, the most successful method to control fungal spread is the application of fungicides, which are costly and have a negative impact on the environment, favor a selection of pathogen resistance and, in severe cases, are ineffective [16]. In this context, understanding the molecular basis of the soybean defense against fungal infection and growth, identifying genes involved in susceptible or resistant response and characterizing their individual roles are key steps for engineering durable and quantitative disease resistance. Therefore, genetic transformation represents a powerful tool for functional studies.

Many studies have implicated a role for soybean WRKY transcription factors in the response to *P. pachyrhizi* infection [17-22]. *WRKY* genes might regulate the expression of defense genes, modulating immediate downstream target genes or activating/repressing other transcriptional factors [23].

WRKY transcription factors comprise one of the largest families of regulatory proteins in plants. Previous studies have identified 72 *WRKY*-encoding genes in *Arabidopsis* [24], approximately 100 members in rice [25-28], 104 in poplar [29], 86 in *Brachypodium distachyon* [30], 80 in grape [31] and 116 and 102 genes in two different species of cotton [32]. A genome-wide analysis in primitive eukaryotes [33] revealed the widespread occurrence of WRKY proteins.

The most prominent feature of these proteins is the WRKY domain, which is a highly conserved 60 amino acid region hallmarked by the heptapeptide WRKYGQK followed by a C_2_H_2_- or C_2_HC zinc-finger motif. As deduced from the results of a nuclear magnetic resonance analysis of a WRKY domain of *At*WRKY4, the conserved WRKYGQK sequence is directly involved in DNA binding [34], but the zinc finger motif is also required [35]. Most of the well-characterized WRKY proteins bind to the W-box element (C/T)TGAC(C/T) in the promoter region of the target genes [36]. The specificity of the binding site is partly dependent on the DNA sequences adjacent to the W-box core, and the involvement of WRKY factors in protein complexes might be the major criteria in determining promoter selectivity [37].

The identification of 64 *WRKY* genes expressed in various soybean tissues and in response to abiotic stress was previously assessed using RT-PCR [38]. However, due to the unavailability of the complete soybean genome sequence at that time, the number of members of this gene family was underrepresented. Yin et al. [39] identified 133 WRKY members in soybean genome. Now a day, several databases for soybean genome analysis are publicly available. PlantTFDB [40] SoyDB [41] and SoyTFKB [42] are transcription factor databases which contain valuable information, including protein sequence, protein domains, predicted tertiary structures and links to external databases. However, despite the usefulness, these databases have performed systematic annotations resulting in different numbers of soybean WRKY transcription factors and some incorrect gene models. So, until now, there is no a comprehensive curate list of soybean *WRKY* genes. Besides, there is inconsistent nomenclature for soybean WRKY members in the literature. The Phytozome database (http://www.phytozome.org**)** assigns names from *Arabidopsis* orthologs, while Zhou et al. [38] identified 64 soybean *WRKY* genes (deposited in http://www.ncbi.nlm.nih.gov/) and randomly assigned a number to each gene. Moreover, studies of the individual genes [43,44] have assigned numbers different from those proposed by Zhou et al. [38]. The present study reports a genome-wide annotation of the WRKY family in soybean and a functional analysis of some genes involved in response to *P. pachyrhizi* infection.

## Results

### Annotation and *in silico* characterization

In total, 182 potentially *WRKY*-encoding genes were identified and annotated in the present work (Table 1 and Additional file 1). Additionally, a total of 33 putative WRKY pseudogenes were found (Additional file 2). Some of them were identified in our search and other ones were previously described in the USM data set [45]. Transcripts for 152 annotated *WRKY* genes were detected on SoyBase EST database (http://soybase.org/) and/or on five global expression experiments: SuperSAGE of soybean leaves 12, 24 and 48 hours after inoculation (hai) of *P. pachyrhizi* [46], RNA-Seq of microdissected lesions 10 days after inoculation of *P. pachyrhizi*, two different microarrays of leaves 12 and 120 hai of *P. pachyrhizi* (available in the current literature) and RNA-Seq expression data of healthy plants in different developmental stages [47], available at SoyBase [48]. The *GmWRKY* genes were distributed over the 20 soybean chromosomes with protein sequences ranging from 121 to 1,356 amino acids in length (Table 1 and Additional file 1). There was an average of 9.1 *WRKY* genes per chromosome, with the highest number of genes (15 genes) located on chromosome 6.

**Table 1:** **Annotation of** ***Glycine max*** **WRKY transcription factors (Choromosome 1 to 3)**

**Chr**	**Gene ID** ^**a**^	**Name** ^**b**^	**Alternative transcripts**	**CDS (pb)**	**Protein (aa)**	**Groups** ^**c**^	**Expression**	**Soybase**	**Domain modifications**
	**(Phytozome)**						**Confirmed** ^**d**^	**EST ID**	
1	Glyma01g05050	*GmWRKY3*	1	1530	510	IIb	+	-	
1	Glyma01g06550	*GmWRKY9*	1	1368	456	I	+	EU019557.1	
1	Glyma01g06870	*GmWRKY28*	2	894	298	IIc	+	CA938308.1	
1	Glyma01g31921	*GmWRKY5*	2	1524	508	I	+	EU019554.1	WRKYGQK → WRKYG**E**K (N-terminal)
1	Glyma01g39600	*GmWRKY35*	2	966	322	IId	+	BG651351.1	
1	Glyma01g43130	*GmWRKY65*	1	738	246	IIe	+	-	CX_(N)_CX_(N)_HXH/C → CX_(N)_CX_(N)_HX**D**
1	Glyma01g43420	*GmWRKY12*	1	969	323	III	+	EU019558.1	
2	Glyma02g01031	*GmWRKY66*	1	1455	485	IIb	-	-	
2	Glyma02g01420	*GmWRKY67*	1	963	321	IIc	+	BT096212.1	
2	Glyma02g02430*	*GmWRKY68*	2	1443	481	IIb	-	-	
2	Glyma02g12490	*GmWRKY69*	1	1368	456	I	+	FK022538.1	
2	Glyma02g12830	*GmWRKY32*	1	882	294	IIc	+	BM527576.1	
2	Glyma02g15920	*GmWRKY22*	4	1068	355	IId	+	AK244154.1	
2	Glyma02g36510	*GmWRKY70*	1	1518	506	I	+	FG988660.1	
2	Glyma02g39870	*GmWRKY39*	1	1743	581	I	+	BM188894.1	
2	Glyma02g45530	*GmWRKY71*	1	1014	338	IIc	+	BE020472.1	
2	Glyma02g46280	*GmWRKY72*	2	1206	402	IIb	-	-	
2	Glyma02g46690	*GmWRKY73*	2	1767	589	I	+	BG789786.1	
2	Glyma02g47650	*GmWRKY74*	1	1524	508	I	+	CO984087.1	
3	Glyma03g00460	*GmWRKY75*	1	816	272	III	+	BT095645.1	
3	Glyma03g05220	*GmWRKY76*	1	1524	508	I	+	EV272592.1	WRKYGQK → WRKYG**E**K (N-terminal)
3	Glyma03g25770	*GmWRKY77*	1	717	239	IIc	+	EV274902.1	
3	Glyma03g31630	*GmWRKY15*	2	1026	342	IId	+	CD397604.1	
3	Glyma03g33376	*GmWRKY29*	2	1347	449	I	+	EU019569.1	
3	Glyma03g37870	*GmWRKY41*	1	762	254	IIe	+	EU019577.1	
3	Glyma03g37940	*GmWRKY51*	1	864	288	IIc	+	BT098285.1	
3	Glyma03g38360	*GmWRKY78*	2	1626	542	IIb	+	DB956313.1	
3	Glyma03g41750	*GmWRKY43*	1	1089	363	III	+	EU019579.1	

^a^Reannotated genes with original sequences containing wrong start\stop codons are marked with (*).

^b^The names *Gm*WRKY1-64 are given according to Zhou et al. [38]; *Gm*WRKY65-182 are given according to the chromosome order.

^c^The classification according to Eugelm et al. [24].

^d^The expression confirmation according to SoyBase ESTs, RNA-Seq analysis (*in silico* analysis) and RNA-Seq of ASR lesion microdissection (experimental analysis).

The proteins were assigned to three major groups and subgroups in accordance with Eugelm et al. [24]. Group I, II and III contained 31, 126 and 25 soybean *WRKY* genes, respectively (Table 1 and Additional file 1). A total of 13, 33, 42, 16 and 22 proteins were assigned to subgroups IIa, IIb, IIc, IId and IIe, respectively.

Although the WRKYGQK signature was highly conserved in the soybean WRKYs, 15 proteins with amino acid substitutions in the signature of the C-terminal domain were identified. These variant proteins were distributed among all groups, except subgroup IId. WRKYGKK was the most common variant and was shared by 11 genes. Other atypical sequences, such as WRKYGEK, WRKYEDK, WKKYGQK, CRKYGQK and WHQYGLK, occurred in single proteins. Nine WRKY proteins contained incomplete and/or amino acid substitutions in the zinc-finger sequence (Table 1 and Additional file 1). Some of these proteins contained patterns of zinc-finger motifs that have not been reported in the literature. Expression was detected for nine genes presenting modifications in the WRKY signature and for six genes with modifications in the zinc-finger motif, indicating that these genes might be functional. Moreover, another highly conserved domain, the zinc cluster, was identified upstream of the WRKY domain in IId gene members.

**Figure 1:**
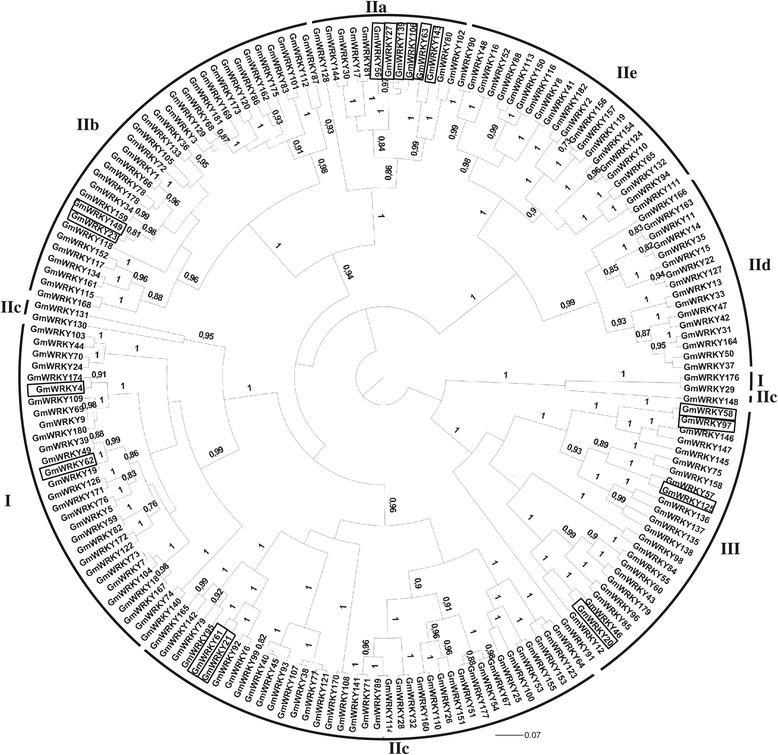
**Dendogram representing the relationship among the soybean WRKY proteins.** The tree was reconstructed using a Bayesian (BA) method. A total of 182 amino acid sequences from *G. max* and 65 sites corresponding to WRKY domain were included in the analysis. The posteriori probability values are labeled above the branches and only values higher than 70% are presented. The groups I, IIa, IIb, IIc, IId, IIe and III are indicated. Differentially expressed genes in response to *P. pachyrhizi* infection are boxed in black.

The phylogenetic approach performed with the WRKY domain sequences confirmed the division of *Gm*WRKY members in the five groups (I, IIa + IIb, IIc, IId + IIe and III) (Figure 1 and Additional file 3). These groups correspond to the WRKY domain classification (groups and subgroups I, IIa, IIb, IIc, IId, IIe and III) that has already been demonstrated in other studies. Genes from Group IIa are closely related with those from Group IIb, while genes from Group IId are closely related with those from Group IIe.

### Gene expression data

An overview of the differential expressed soybean *WRKY* genes that were modulated in response to *P. pachyrhizi* infection is presented in Table 2 and Additional file 4. The expression data were obtained from four global expression experiments: SuperSAGE of leaves 12, 24 and 48 hours after inoculation (hai), RNA-Seq of microdissected lesions 10 days after inoculation and two different microarrays of leaves 12 and 120 hai, available in the current literature [17,22]. Seventy-five genes showed differential expression in at least one experiment, whereas 16 genes showed differential expression in more than one experiment. Genes from groups I, II and III responded to this stress condition.

**Table 2:** **Expression pattern of WRKY encoding-genes under** ***P. pachyrhizi*** **infection** ^**a**^ **(Group I and IIa)**

**Group**	**Gene ID**	**SuperSage - LGE**	**RNA-Seq of lesion LCM** ^**b**^	**Microarray – van de Mortel et al. [** 17 **]** ^**c**^				**Microarray - Schneider et al. [** 22 **]**			
		**Incompatible reaction (PI561356-** ***Rpp1*** **)**	**PI561356 X BRS231**	**Incompatible reaction (PI230970-** ***Rpp2*** **)**		**Compatible reaction (Embrapa48)**		**Compatible reaction (PI462312-** ***Rpp3*** **X Taiwan 80-2)**		**Incompatible reaction (PI462312-** ***Rpp3*** **X Hawaii 94-1)**	
		**Inoculated X Mock**	**Inoculated**	**Inoculated X Mock**		**Inoculated X Mock**		**Inoculated X Mock**		**Inoculated X Mock**	
		**12, 24, 48 h**	**10 days**	**12 h**	**120 h**	**12 h**	**120 h**	**12 h**	**144 h**	**12 h**	**144 h**
I	Glyma03g05220			x	x	x	x				
I	Glyma01g31921			x	x	x	x				
I	Glyma18g44030				x	x	x				
I	Glyma09g41670		x		x	x	x				
I	Glyma18g06360		x	x	x	x	x				
I	Glyma11g29720			x	x	x	x				
I	Glyma14g38010			x	x	x	x				
I	Glyma02g39870			x	x	x	x				
I	Glyma09g38581		x								
I	Glyma04g12830			x	x	x	x				
I	Glyma06g47880			x	x	x	x				
I	Glyma08g43770			x	x	x	x				
I	Glyma18g09040			x	x	x	x				
I	Glyma07g35381		x								
I	Glyma18g49830			x		x					
I	Glyma08g26230			x		x			x		
IIa	Glyma04g06470		x								
IIa	Glyma17g33920			x	x	x	x				x
IIa	Glyma14g11920			x	x	x	x	x	x	x	
IIa	**Glyma15g00570**	**x**		**x**	**x**	**x**	**x**				**x**
IIa	**Glyma13g44730**			**x**	**x**	**x**	**x**				
IIa	**Glyma08g23380**			**x**	**x**	**x**	**x**		**x**		**x**
IIa	**Glyma07g02630**			**x**	**x**	**x**	**x**				**x**
IIa	Glyma17g33891			x	x	x	x				
IIa	Glyma14g11960	x	x	x		x	x				

^a^The expression data were obtained from four global expression experiments: SuperSAGE available at www.lge.ibi.unicamp.br/soja/, RNA-Seq of microdissected lesions and two different microarrays available in the current literature. The x denotes significant differences (p < 0.05). The genes indicated in bold were used in further analyses. The genes were ordered according to the clustering analysis.

^b^LCM: laser-capture microdissection.

^c^Some probes hybridized with more than one gene.

Some of the genes that presented differential expression profiles in response to the fungus were randomly selected from each classification group for more detailed analyses. *GmWRKY27* (Glyma15g00570) and *GmWRKY125* (Glyma09g41050) were differentially expressed in three of the four experiments, while *GmWRKY56* (Glyma08g23380), *GmWRKY106* (Glyma07g02630) and *GmWRKY20* (Glyma08g02580) in the two microarrays. *GmWRKY139* (Glyma13g44730), *GmWRKY46* (Glyma05g36970), *GmWRKY57* (Glyma18g44560) were also analyzed because they were closely related to at least one of the genes evaluated above. Interestingly, none of these genes was expressed in rust infection lesions at ten days after fungus inoculation (RNA-Seq).

**Figure 2:**
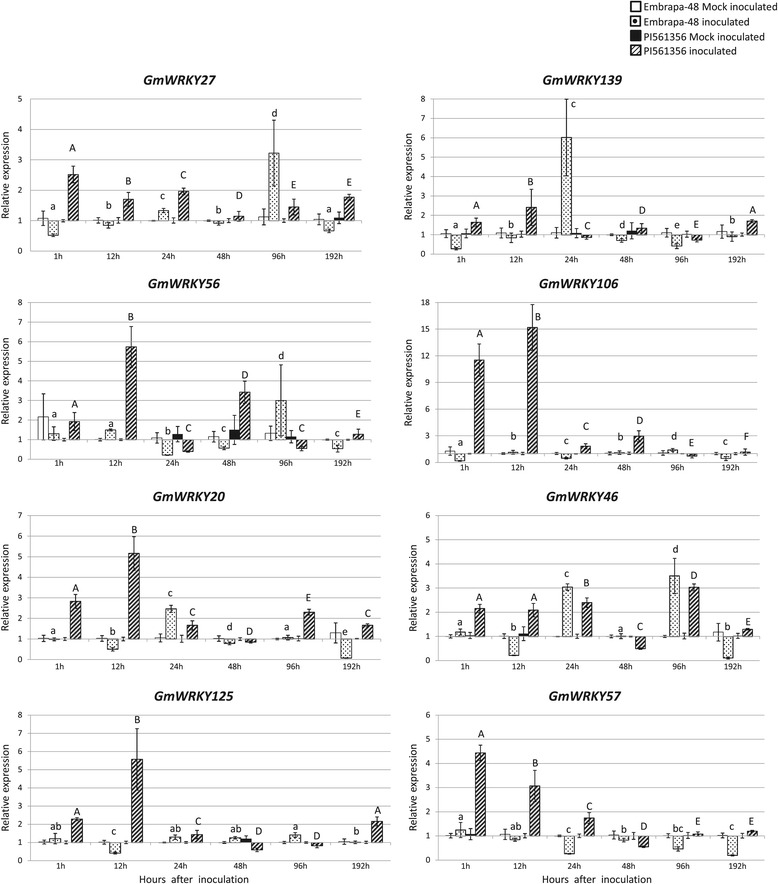
**Expression patterns of** ***WRKY*** **genes in leaves of three-week-old soybean plants infected with** ***P. pachyrizi.*** The gene response in susceptible (Embrapa-48) and resistant (PI 561356) genotypes during *P. pachyrizi* infection (inoculated) was evaluated using RT-qPCR. Mock-inoculated plants were used as a control. The values (mean ± SD) were calculated based on three biological replicates and four technical replicates. Multifactorial analysis of three factors (genotype, treatment and time) was highly significant: *GmWRKY57*, *GmWRKY27*, *GmWRKY125*, *GmWRKY20* and *GmWRKY46* p = 0.0001; *GmWRKY139* p = 0.0265; *GmWRKY56* p = 0.0003. The means indicated with the same letters in the same cultivar and treatment did not differ significantly (Tukey’s multiple comparison test, p < 0.05). Lower case letters were used to identify differences among inoculated Embrapa-48 plants and capital letters were used to identify differences among inoculated PI561356 plants. F-Box protein and metalloprotease reference genes were used as internal controls to normalize the amount of mRNA present in each sample. Transcript levels of *WRKY* genes present in mock-inoculated plants were used to calculate transcript accumulation in the inoculated plants.

The differential expression of these genes was confirmed using RT-qPCR. The transcript levels during the course of fungus infection in a resistant genotype (PI561356) and in a susceptible genotype (Embrapa-48) were compared with those in the mock-inoculated plants (Figure 2).

The interaction among the genotypes, time-course and pathogen presence was highly significant (p < 0.0001). In the inoculated plants, the eight genes showed early expression in PI561356 (resistant) compared with Embrapa 48 (susceptible). In the Embrapa 48, the expression peaks were higher at 24 and**/**or 96 hai, while in PI561356, these peaks varied from one to 24 hai. Furthermore, *GmWRKY56*, *GmWRKY106*, *GmWRKY20* and *GmWRKY125* presented a stronger response in the resistant genotype. Interestingly, the homologous genes (*GmWRKY27* and *GmWRKY139*, *GmWRKY125* and *GmWRKY57*) did not overlap with their expression peaks in the resistant genotype. *GmWRKY27* and *GmWRKY57* showed higher expression levels at one hai followed by a decrease in expression, whereas *GmWRKY139* and *GmWRKY125* presented higher transcript levels at 12 hai.

### *GmWRKY27* overexpression and silencing in soybean plants

*GmWRKY27* was selected for further functional characterization because it was one of the genes that showed differential expression in different experiments. Furthermore, it was also shown that this gene is involved in different abiotic stresses [38]. To determine the functional role of the *GmWRKY27* in response to *P. pachyrhizi* infection, soybean somatic embryos were transformed to obtain gene overexpression and silencing. In the overexpression experiments, GFP expression was detected in hygromycin-resistant globular embryos (Additional file 5A and B). The histodifferentiated embryos of nine independent transgenic lines (seven from Biobalistic and two from bombardment/*Agrobacterium*) were obtained. The presence of the T-DNA in the embryo genomes was confirmed using PCR, and the *GmWRKY27* expression was significantly higher in the embryos of the four independent transgenic lines (Additional file 5C). However, the development of transgenic embryos overexpressing *GmWRKY27* was not successful. As a consequence, those embryos were not able to develop into plants.

For gene silencing, a vector carrying a 176-bp inverted-repeat fragment sequence from *GmWRKY27* was constructed. This fragment shared 83% similarity with the homologous region of *GmWRKY139* and 70% and 67% similarity with *GmWRKY56* and *GmWRKY106* respectively. These data confirm the close relationship among the genes, which was also observed in the phylogenetic analysis (Figure 1). This high sequence similarity suggests that the silencing construct would target the four genes.

**Figure 3:**
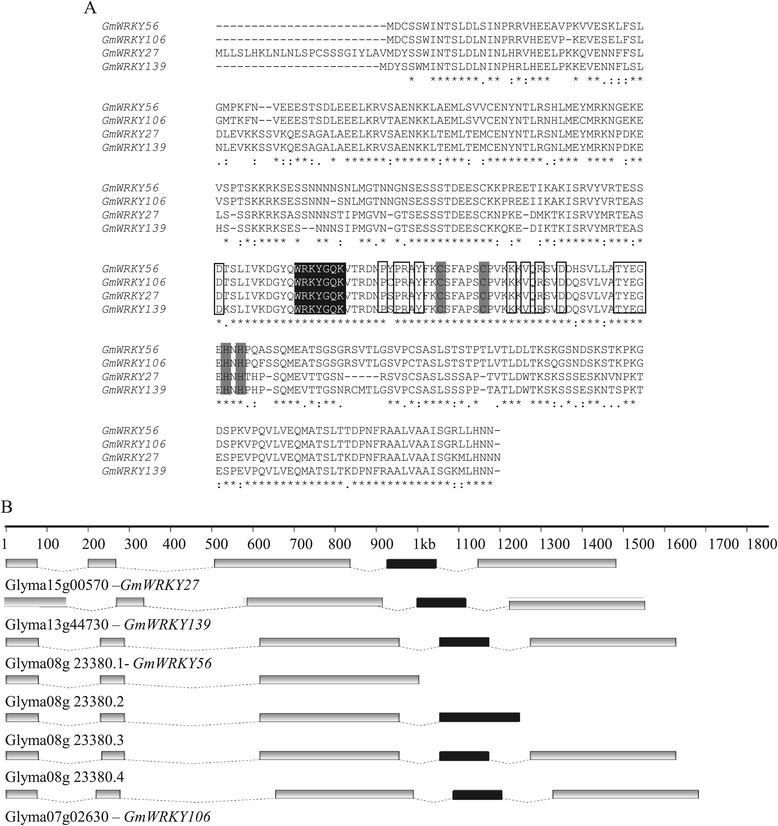
**Amino acid alignment, conserved residues and structure of the four soybean** ***WRKY*** **genes. (A)** Amino acid alignment and identification of conserved residues. The conserved WRKY amino acid signature and the amino acid forming the zinc-finger motif are highlighted in black and gray, respectively. Other conserved amino acids are boxed in black. Multiple sequence alignment was performed using CLUSTAL W 2.1. Highly conserved residues are indicated by (*), strongly similar by (:) and weakly similar by (.). **(B)** Structure of *WRKY-*encoding genes. Glyma08gg23380.1, Glyma08gg23380.2, Glyma08gg23380.3 and Glyma08gg23380.4 are alternative transcripts of Glyma08gg23380. The gray boxes represent exons and the black boxes indicate the exons that contain the WRKY domain. The dotted lines represent introns.

A more detailed structural analysis of the four homologous genes showed that the WRKYGQK signature, zinc-finger motif and other residues in the sequences were highly conserved among the four corresponding proteins (Figure 3A). The sequence identity of the complete proteins varied from 66% to 94% (Table 3). The four soybean genes were putative orthologs of *AtWRKY40, AtWRKY18* and *AtWRKY60* Arabidopsis genes, as shown in the phylogenetic tree (Additional file 3). The gene structure of *GmWRKY27*, *GmWRKY139, GmWRKY56* and *GmWRKY106* was similar, with the WRKY domain present in the fourth exon (Figure 3B). Interestingly, *GmWRKY56* had four alternative transcripts, and one of the transcripts lacked the WRKY domain.

**Table 3:** **Identity percentage (%) among the sequences of the four soybean and three** ***Arabidopsis WRKY***

	***GmWRKY139***	***GmWRKY56***	***GmWRKY106***	**AtWRKY40**	**AtWRKY18**	**AtWRKY60**
*GmWRKY27*	83,6	69,7	66,7	59,4	42,2	36
*GmWRKY139*		75,72	76,8	53,4	46,77	39,16
*GmWRKY56*			94,6	49,52	45,05	39
*GmWRKY106*				48,9	46,94	37,94

**Figure 4:**
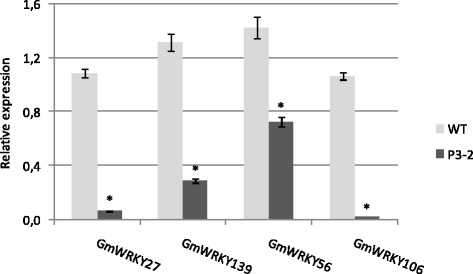
**Expression levels (RT-qPCR) of the soybean-silenced transgenic line for the four** ***WRKY*** **genes.** Expression levels of the four *WRKY* genes in a wild-type (wt) soybean plants and in a transgenic soybean line P3-2. F-Box protein and metalloprotease reference genes were used as internal controls to normalize the amount of mRNA present in each sample. Transcript levels of *WRKY* genes present in the wild type were used to calibrate transcript amounts in P3-2. *Means are significantly different in the wild type and P3-2 plants (Student’s *t*-test, p < 0.05).

Two independent transgenic lines (cultivar BRSMG 68 Vencedora) carrying the silencing construct were obtained. The molecular analysis revealed that one of the repeats (176-bp fragment) was eliminated from the first line. Therefore, the post-transcriptional silencing was not triggered, which was confirmed using RT-qPCR (data not shown). In the second transgenic line (P3-2) the complete cassette was successfully integrated (data not shown). As anticipated, the results from the RT-qPCR analysis showed that the expression of the four homologous genes was significantly reduced (Figure 4). The transgenic line exhibited no major phenotypic alterations.

### The silenced line was shown to be more susceptible to *P. pachyrhizi*

**Figure 5:**
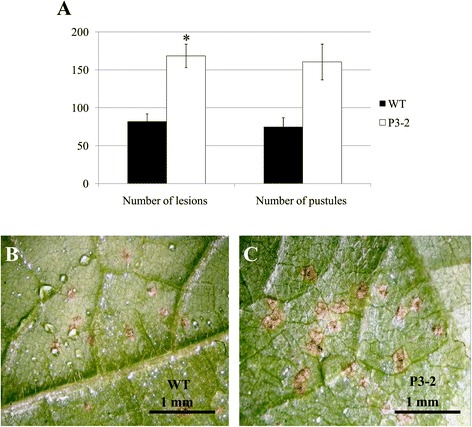
***P. pachyrhizi*** **development on the detached leaves at 12 days after inoculation.** Three detached leaves of each one transgenic line and two wild-type plants were inoculated with 10^5^/mL spore suspension and incubated at 20°C. **(A)** Two infection parameters were evaluated: number of lesions and number of pustules. *Means are significantly different in leaves of wild type (wt) and transgenic soybean line P3-2. (Student’s *t*-test, p < 0.05). **(B)** Low number of tan-colored lesions and pustules under stereomicroscope in a leaf of wild-type (wt) plant. **(C)** High number of tan-colored lesions and pustules under stereomicroscope in a leaf of transgenic soybean line P3-2 with suppression of the four *WRKY*s.

A detached leaf assay was performed to confirm the involvement of *GmWRKY27*, *GmWRKY139*, *GmWRKY56* and *GmWRKY106* in the soybean response to *P. pachyrhizi* infection. As previously described, detached leaf and intact plant bioassays revealed a high correlation [49]. In the present study, “tan” lesions could be observed on all detached leaves of both transgenic and wild type samples at 12 days after *P. pachyrhizi* inoculation. However, the number of lesions was significantly higher in the leaves of the transgenic line (Figure 5). No visible differences were observed concerning the appearance of the lesions and pustule formation or eruption (data not shown).

## Discussion

### Soybean *WRKY* genes

Whole genome sequencing [50] has facilitated the accurate annotation of soybean gene families. In this study, we present the annotation of 182 WRKY transcription factors in soybean. The transcripts of 152 genes were detected, suggesting they can be expressed at the protein level; however, specific conditions might be necessary for the successful transcription of the remaining genes.

As discussed before, there is inconsistent nomenclature for soybean WRKY members in the literature. To unify the terminology, we proposed a nomenclature based on the previously described *WRKY*-encoding genes [38], with some modifications. Data from sequence comparisons have shown that *GmWRKY18* and *GmWRKY35* is the same gene. In addition, *GmWRKY3* does not exist in the soybean genome; indeed, this sequence represents a chimeric transcript produced through *trans*-splicing between N-terminal and C-terminal sequences from Glyma02g46690 and Glyma14g01980, respectively. The remaining 118 genes were numbered according to the order of the chromosomes (Table 1 and Additional file 1).

More *WRKY* genes have been identified in soybean than in other species, such as rice, *Arabidopsis*, cotton, grape and *B. distachyon* [24-28]. The duplication events have been greatly over-retained, specifically in the case of transcription factors [51]. Thus, functional redundancy is a common feature in plant species. However, homologous genes might diverge in function providing a source of evolutionary novelty [52].

The phylogenetic approach used in this study allowed the division of the soybean *WRKY* genes in the five groups previously reported [26,53,54].

In soybean, the members of group I contained domains with a C_2_H_2_-type zinc-finger motif. The same characteristic is observed in *Arabidopsis*, while in rice, the WRKY domains of group I members include two types of zinc-finger motifs: C_2_H_2_ and C_2_HC [25,27].

**Figure 6:**
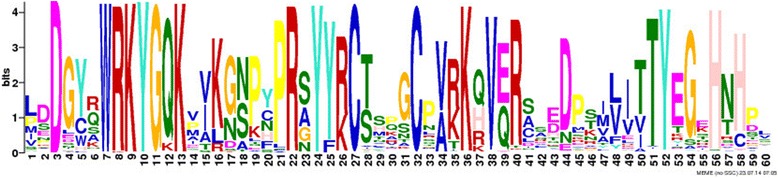
**Conservation analysis of the consensus sequence of the WRKYGQK domain.** Analysis of the 182 soybean *WRKY* genes identified was performed using the MEME suite. The overall height in each stack indicates the sequence conservation at each position. The height of each residue letter is proportional to the relative frequency of the corresponding residue. Amino acids are colored according to their chemical properties: green for polar, non-charged, non-aliphatic residues (NQST), magenta for the most acidic residues (DE), blue for the most hydrophobic residues (A, C, F, I, L, V, W and M), red for positively charged residues (KR), pink for histidine (H), orange for glycine (G), yellow for proline (P) and turquoise for tyrosine (Y).

Although the WRKYGQK signature was highly conserved among soybean WRKY proteins, as illustrated in Figure 6, variation was identified in 21 genes. Zhou et al. [38] previously showed that *GmWRKY6* (Glyma08g15050) and *GmWRKY21* (Glyma04g39650) contain the variant WRKYGKK rather than the conserved WRKYGQK motif. Slight variations in this region have also been reported in *Arabidopsis*, rice, tobacco, barley, canola and sunflower [25,26,55-58]. Compared with Arabidopsis, which contains four WRKYGKK variants, the number of genes with a modified WRKYGQK motif is greater in soybean.

Some unusual *GmWRKY*-encoding genes (i.e., containing a modified WRKY signature and/or zinc-finger motif) produced mRNA (Table 2 and Additional file 4). Further analyses are necessary to determine whether these genes function as transcription factors or if they induce post-transcriptional regulation through RNAi, as previously suggested [23]. Variant proteins might have abolished or decreased capacities to bind to the W-box [35,37]. It has been suggested that WRKY proteins without the canonical WRKYGQK motif might have different binding sites [37,56], target genes and possibly divergent roles [57].

### Functional analysis

Despite the fact that the identification or prediction of many *WRKY* genes from different species has been previously achieved, only a small number of these have been functionally characterized. Information concerning the role of soybean genes (Glyma13g00380-*GmWRKY13*, Glyma04g39650-*GmWRKY21*, Glyma10g01450-*GmWRKY54* and Glyma18g44560-*GmWRKY57*) during abiotic stress has been based on data obtained from heterologous expression systems [38,43]. The data from expression analyses [17,44] or using transient gene silencing [59] supports a role for the *WRKY* genes in response to biotic stresses. Studies concerning global expression profiling have demonstrated the importance of *WRKY*-encoding genes in transcriptional reprogramming during *P. pachyrhizi* infection in soybean plants [17-22].

To determine which soybean *WRKY* genes are involved in plant defense against *P. pachyrhizi* infection, we performed a series of analyses to examine their expression patterns after infection. We initially compared the microarray data available in the literature [17,22] with the results obtained from two additional experiments: SuperSAGE and RNA-Seq. Many genes were differentially expressed in only one library, while a few of them showed differential expression in more than one library. The modulation in the transcript levels of eight genes was validated, showing the reliability of data mining. The similar expression patterns in response to *P. pachyrhizi* infection was observed among closely related genes (Figure 1), such as *GmWRKY61* (Glyma06g15220) and *GmWRKY21* (Glyma04g39650), *GmWRKY143* (Glyma14g11920) and *GmWRKY63* (Glyma17g33920), *GmWRKY106* and *GmWRKY56*, *GmWRKY58* (Glyma04g40130) and *GmWRKY97* (Glyma06g14720). This similar expression pattern suggests that these genes might share similar functions in disease resistance. The redundant function of *GmWRKY* genes might be beneficial in protecting the cell or organism under various stress conditions and eliciting multiple pathways that lead to the wide array of physiological responses that occur following pathogen infections [60].

Global expression data have suggested that the timing and the degree of induction of the defense pathway are determinants for the induction of soybean resistance to *P. pachyrhizi* [17,20,22,60]. In our study, the induced expression of *GmWRKY20, GmWRKY27, GmWRKY46, GmWRKY57*, *GmWRKY56, GmWRKY106, GmWRKY125* and *GmWRKY139* in response to *P. pachyrhizi* was earlier and**/**or stronger in the resistant genotype. The expression of most genes analyzed peaked at 12 hai in the resistant genotype; therefore, we propose that these genes might be involved in non-specific defense responses. Van de Mortel et al. [17] and Schneider et al. [22] reported that *P. pachyrhizi* infections induce biphasic global expression. Gene expression initially peaked at 12 hai, which corresponded with the early infection processes of appressoria formation and epidermal cell penetration. The authors suggested that this peak corresponded to a non-specific defense response similar to pathogen-triggered immunity. A second phase of gene expression, which began at 72 hai and continued until 288 hai, is coincident with haustoria formation and effector protein secretion. The authors suggested that this response is consistent with the activation of *RPP2* and *RPP3*-mediated resistance. It has been shown that gene expression is rapid and increased in the incompatible interaction [17,18,22].

The closely related genes *GmWRKY27*, *GmWRKY139*, *GmWRKY56* and *GmWRKY106* are putative orthologues of *AtWRKY40, AtWRKY18* and *At*WRKY60 *Arabidopsis* genes. In both species, these genes were classified into group IIa. The three *Arabidopsis* WRKYs are involved in stress responses, which include resistance against the bacteria *Pseudomonas syringae* and fungus *Botrytis cinerea* [61,62]. *At*WRKY18 is a salicylic acid-induced gene that positively regulates SAR [63,64] and modulates PR gene expression; *At*W*RKY18* overexpression increases resistance to *P. syringae* [65]. *At*WRKY40 and *At*WRKY60 proteins antagonize *At*WRKY18 during *P. syringae* infection. The gain or loss of gene function in single, double or triple combination mutants resulted in increased susceptibility to *B. cinerea* [61]. Some rice, barley and *Brassica napus* WRKY members from group IIa are also involved in the response to fungal and bacterial pathogens, as demonstrated using expression studies. O*sWRKY62* and *OsWRKY76* are upregulated in *Magnaporthe grisea* infected-leaves *and* downregulated in *Xanthomonas oryzae*-inoculated leaves [66]. *HvWRKY1* and *HvWRKY2* play an important role in response to *Blumeria graminis* infection [55], and *BnWRKY18 and BnWRKY40* play a role in the response to *Sclerotinia sclerotiorum* and *Alternaria brassicae* infections [57].

Most available information concerning soybean gene function is based on data obtained from heterologous expression systems. However, as the activity of many proteins frequently depends on specific interactions that are only found in homologous backgrounds, the present study was based on a homologous expression system. An RNA interference approach was used for the silencing of four soybean homologous genes (*GmWRKY27*, *GmWRKY139*, *GmWRKY56* and *GmWRKY106*). The quadruple silencing is an advantage because a single knockout of transcription factors rarely results in altered phenotypes due to functional redundancy among closely related members [65]. The transgenic RNAi line used in this study generated a significant reduction in the transcript levels of the four target genes. When infected with *P. pachyrhizi*, the transgenic line showed increased susceptibility to the fungus. Taken together, the results strongly suggest that at least one of the four genes might be involved in the soybean resistance phenotype.

Pandey et al. [59] silenced 64 soybean WRKYs individually using virus-induced gene silencing (VIGS) to test their involvement in *Rpp2-*mediated resistance against *P. pachyrhizi* infection. Three of these genes (*GmWRKY45*, *GmWRKY40* and *GmWRKY36*) compromised the resistance phenotype when silenced. Phenotypic alterations were not evidenced when *GmWRKY56* and *GmWRKY106* genes were individually silenced*.* However, in the present study, an increased susceptibility to *P. pachyrhizi* infection was observed in the quadruple-silenced (*GmWRKY27*, *GmWRKY139, GmWRKY56* and *GmWRKY106*) line, suggesting that this phenotype is a consequence of *GmWRKY27* and/or *GmWRKY139* silencing. Moreover, the four genes analyzed in this study could also play a synergistic role in the pathogenic defense response.

A previous study showed that *GmWRKY27* is also strongly induced under conditions of drought and salt stress in the soybean [38]. Altogether, these data suggest that this gene is probably involved in a non-specific response that occurs upstream of biotic and abiotic stress defense routes, in contrast with the specific *Rpp2-*response of the genes identified by Pandey et al. [59] in response to the fungal infection.

*GmWRKY27* was selected for use in the overexpression study. Histodifferentiated embryos overexpressing this gene were obtained from four independent transformation experiments. However, the plants were not recovered. The most likely explanation is that the constitutive overexpression of the *GmWRKY27* might affect the regeneration of plants. The use of constitutive promoters in investigation of genes whose constant overexpression has deleterious effects on the plant is a major limitation [67]. Chen and Chen [65] reported that high levels of *At*WRKY18 cause severe abnormalities in plant growth. Even at moderate levels, the individual or combinatorial overexpression of *AtWRKY18*, *AtWRKY40* and *AtWRKY60* leads to the development of smaller plants or death shortly after germination [61].

The deleterious effect of the excessive production of these WRKYs during plant growth suggested that the expression of this gene might require proper regulation during the activation of plant defense responses. However, in healthy plants, the expression of these genes is negatively regulated, as demonstrated by Chen and Chen [65] for the *AtWRKY18*.

To a certain extent, the lethality problems observed in this study could be partially overcome using tissue-specific, developmentally regulated or inducible promoters. Although the number of tissue-specific promoters has increased in recent years, soybean leaf-specific promoters are still unavailable.

## Conclusions

In the present study, 182 WRKY transcription factors were annotated in soybean. Seventy-five genes were identified as involved in the soybean response to *P. pachyrhizi* infection based on transcriptional regulation*.* The participation of four genes in response to pathogen infection was demonstrated using an RNAi approach. Further investigations are required to provide clues regarding the functions of the individual genes. The results contribute to the elucidation of gene function and suggest the manipulation of WRKYs as a strategy to increase fungal resistance in soybean plants.

## Methods

### Database search and sequence annotation

To search for *Glycine max* (*Gm*) *WRKY* transcription factor we use two different approaches as follow: first we downloaded soybean proteome from Phytozome (http://www.phytozome.org**)** and SoyBase (http://soybase.org/) databases to perform a Batch BLAST using BLASTALL software [68]. The WRKY domains previously identified in *Arabidopsis* [40], poplar [40] and soybean [40-42,45] genomes were checked on the SMART Web Site and were used as queries to perform tblastp (e-value cut off of 10) searches. After doing Batch BLAST searches we checked for soybean *WRKY* genes in PlantTFDB (http://planttfdb.cbi.pku.edu.cn/) transcription factor database and USM data set [45].

Additionally, we used the coding sequences (CDS) to perform blast searches against the Phytozome database (www.phytozome.org**)** and PLAZA (http://bioinformatics.psb.ugent.be/plaza/) to retrieve any additional *WRKY* genes. The Phytozome database was also used to obtain the gene structures. The automated *WRKY*-predicted gene sequences that contained incorrect gene models (wrong start/stop codons or truncated proteins) were reannotated using GENSCAN [69] and FEGENESH [70] predictors, considering 2, 5 or 10-kb DNA sequences obtained from Gbrowse. The sequences were aligned with ClustalX v2.1 [71], and the domains manually examined. The sequences without conserved WRKYGQK domain signatures were discarded. The degree of conservation of the WRKYGQK and zinc finger domains was analyzed using the MEME suite **(**http://meme.sdsc.edu/meme/). The annotated genes were classified in groups and subgroups proposed consistent with the methods of Eugelm et al. [24] for *Arabidopsis thaliana.* A nomenclature for the *WRKY*-encoding genes identified in this work was adopted, according to the order of the chromosomes. The structures of the four soybean *WRKY*-encoding genes selected to the functional analysis and their alternative transcripts were analyzed using Fancy Gene v1.4 [72].

### Soybean WRKY relationships

In order to classify the soybean *WRKY* genes identified, a phylogenetic approach was performed with two dataset: the first one contained only soybean WRKY sequences and the second included also *Arabidopsis thaliana* and *Populus trichocarpa* WRKY sequences, downloaded from PlantTFDB database. The multiple sequences alignments were performed with MUSCLE software [73], implemented in MEGA5 (Molecular Evolutionary analysis) software [74]. Phylogenetic analyses were conducted with WRKY domain sequences using Bayesian approach implemented in BEAST1.7 software [75]. The best-fit model of protein evolution was determined using ProTest [76], which selected the JTT model for protein matrix substitution. The Yule tree was selected as a tree prior for Bayesian analysis and 30.000.000 generations were performed with Markov Chain Monte Carlo (MCMC) algorithms. The trees were visualized and edited in FigTree v1.3.1 software [77].

### Gene expression data mining

The *Gm*WRKY CDSs were searched into RNA-Seq expression data [47] which is available at SoyBase [48]. In addition, the expression profiles of the *WRKY* genes that were modulated in response to *P. pachyrhizi* infection were obtained from four different sources. The reaction of soybean plants to rust infection of the first three experiments was assessed by the inoculation of *P. pachyrhizi* spores collected in the field into plants maintained under greenhouse conditions at Embrapa Soja, Londrina, PR, Brazil. The sources used to obtain the expression profiles of the *WRKY* genes are described:

a) SuperSAGE: The libraries were constructed using the leaves of a soybean resistant genotype (PI561356), which carries the *Rpp1* resistance gene, infected with *P. pachyrhizi* vs. uninfected leaves (mock inoculation/control) collected at 12, 24 and 48 hours after inoculation (hai). A Plant RNeasy kit (Qiagen) was used for RNA extraction and equal amounts of RNA from each sample were used to construct the RNA pools. The libraries (inoculated and mock) were constructed at GenXPro GmbH (Frankfurt, Germany) using previously described methods [78] and subsequently sequenced using the Illumina Genome Analyzer IIx. The SuperSAGE tags were analyzed using the DiscoverySpace software v.4.01 [79] to identify unique (unitags) and differentially expressed tags (p ≤ 0.05). The libraries were constructed as part of the GenoSoja project (Brazilian Soybean Genome Consortium), and the results are available in the LGE (Laboratório de Genômica e Expressão, UNICAMP) Soybean Genome database [80] for members of the consortium.

b) RNA-Seq of lesion LCM (Laser Capture Microdissection): foliar segments (1 cm^2^) containing *P. pachyrhizi* lesions from two soybean resistant (PI561356) and susceptible BRS231 [81] genotypes at the V2 growth stage were collected at 10 days after infection. The leaf segments were immediately fixed on ice in Farmer’s solution [82], dehydrated and embedded on paraffin in accord with the methods of Cai and Lashbrook [83]. Serial sections of 12-μm in thickness were generated using a rotary microtome and transferred to microscope membrane slides. Twenty sections containing a variable number of rust lesions were prepared for each biological replicate/treatment. The PixCell II LCM system (Arcturus) and CapSure Macro LCM (Arcturus) were used to collect the foliar cells within the lesion. Total RNA was extracted using the PicoPure RNA Isolation Kit (Arcturus) from the cells collected at a variable number of infection sites for each biological replicate. The synthesis of cDNA was conducted, and high-performance paired-end (108 bp) sequencing was performed on the Illumina genome analyzer GAAllx. Low-quality RNA-Seq reads were discarded. The reads (a total of 86,301,242) were aligned against the soybean genome, and the corresponding genes were predicted using the TopHat [84] and SOAP2 [85] alignment programs. Gene expression was calculated using the FPKM (fragments per kilobase of exon per million fragments mapped) value [86]. To identify differentially expressed genes, a pair-wise comparison between the FPKM values of both genotypes was performed using a *t*-test at the 99% confidence level. This library was constructed as part of the Biotecsur Consortium and the results are available [87] for members of the consortium.

c) Microarray [17]: The expression o *WRKY* genes in the leaves of the soybean resistant genotype (PI970230), which carries the *Rpp2* gene, and in the soybean susceptible genotype (Embrapa 48) in response to *P. pachyrhizi* infection were compared with that of uninfected leaves (mock inoculation). In the present study, the data obtained at 12 and 120 hai were considered because the highest gene expression was exhibited at these time points. Only the 46 probes previously described as WRKYs were examined. The specificity of probes was analyzed using the SoyBase and Phytozome databases. Probes with *e*-values <0.05 were considered.

d) Microarray [22]: The global expression of the soybean cultivar Ankur (PI462312), which carries the *Rpp3* resistance gene, which was inoculated with avirulent (Hawaii 94-1) and virulent (Taiwan 80-2) isolates of *P. pachyrhizi*, was analyzed. The Affy probe sets were searched using the tools available in the Soybase database. In the present study, only the WRKY probes that hybridized with a single locus in the soybean genome were selected. The data obtained at 12 and 120 hai were considered because the highest gene expression was exhibited at these time points. The genes with a p-value <0.05 were considered as differentially expressed.

### *P. pachyrhizi* bioassay for gene expression analysis

Soybean plants were grown in a pot-based system maintained in greenhouse conditions at 28 ± 1°C under a 16/8 h light/dark cycle with a light intensity of 22.5 μEm^-2^s^-1^ in Embrapa Soja, Londrina, PR, Brazil. The Embrapa-48 genotype, which develops a “tan” lesion [17], was used as the susceptible standard, and the PI561356 genotype, which carries the *Rpp1* resistance gene [88], was used as the resistant standard. ASR isolated from Brazilian fields was maintained in a susceptible cultivar. Spores harvested from leaves exhibiting sporulating uredinia and diluted in distilled water containing 0.05% Tween-20 to a final concentration of 3 × 10^5^ spores/mL. The spore suspension was sprayed onto plantlets at the V2 developmental stage. The same solution without spores was used for the mock inoculation. Subsequently, the water-misted bags were placed over each pot for one day. One trifoliate leaf from each plant was collected at 1, 12, 24, 48, 96 and 192 hai, frozen in liquid nitrogen, and stored at -80°C. Three biological replicates from each genotype/treatment were analyzed.

### Expression pattern analysis using reverse transcription and quantitative real-time PCR (real-time RT-qPCR)

Total RNA was extracted using TRIzol reagent (Invitrogen) and further treated with DNAse (Promega) according to the manufacturer’s instruction. The first-strand cDNAs were obtained using 2 μg of DNA-free RNA using the M-MLV Reverse Transcriptase System (Invitrogen) with a 24-polyVT primer. The RT-qPCR was conducted using a StepOne Applied Biosystems Real-Time cycler™. The PCR-cycling conditions were implemented as follows: 5 min at 94°C, followed by 40 cycles of 10 s at 94°C, 15 s at 60°C and 15 s at 72°C, and a final step of 2 min at 60°C. A melting curve analysis was performed at the end of the PCR run over a range of 55–99°C, increasing the temperature stepwise by 0.1°C every 1 s. Each 25-μL reaction comprised 12.5 μL of diluted DNA template, 1X PCR buffer (Invitrogen), 2.4 mM of MgCl_2_, 0.024 mM of dNTPs, 0.1 μM of each primer, 2.5 μL of SYBR Green (1:100000-Molecular Probes Inc.) and 0.03 U of Platinum Taq DNA polymerase (Invitrogen). The cDNA (1:100) templates were evaluated. All PCR reactions were performed in technical quadruplicates. Reactions lacking templates were used as negative controls.

The PCR reactions were performed using gene-specific primers (Table 4). Primer-pairs designed to amplify F-Box proteins and metalloprotease sequences were used to normalize the amount of mRNA present in each sample. These genes were previously confirmed as good reference genes for the experimental conditions used in the present study [89]. The expression analyses were performed after the comparative quantification of amplified products using the 2^-ΔΔCt^ method [90]. The results were statistically compared using variance analysis with three-factor factorial treatments: genotype, time and pathogen presence. The data were transformed using the weighted least squares method. The means were compared using Tukey’s multiple comparison test.

**Table 4:** **Primer set designed for RT-qPCR**

**Target**	**Orientation**	Tm (°C)*	**Primer Sequence**	**PCR product size (pb)**
*GmWRKY20* transcripts	Forward	60	5′-TTGCAAAGTTCAGAAGTATCTTGTC-3′	264
	Reverse	60	5′-GTGACCTGTTGTAGATCCCATC-3′	
*GmWRKY27* transcripts	Forward	61.69	5′-GATTGTGCATTTGCTAATCATGC-3′	105
	Reverse	59.93	5′- GCTATAGAAACTTCGCCAGAAC-3′	
*GmWRKY46* transcripts	Reverse	60	5′-CAATGCATCATCAACTTCCG-3′	213
	Forward	60	5′-CAAGACCACTTTCACAGCTCAC-3′	
*GmWRKY56* transcripts	Forward	60.06	5′-CACCCATCTGCCTCATCAC-3′	234
	Reverse	59.17	5′-GGAGGCCGAGTCTGTACAAT-3′	
*GmWRKY57* transcripts	Forward	60	5′-TCCCTCAACTTCCTCCAATC-3′	170
	Reverse	60	5′-GGAAGGGTTCAAAGGCATC-3′	
*GmWRKY106* transcripts	Forward	59.72	5′-GGAAATAAAGTTCCACTAAGGATGAC-3′	174
	Reverse	60.57	5′ - CCGAGAATGTGTGCTACAACC-3′	
*GmWRKY125* transcripts	Forward	60	5′-TCTCATCTTCCAATAATTTCCCA-3′	135
	Reverse	60	5′-CATGATGCCTTGGTGAGCTA-3′	
*GmWRKY139* transcripts	Forward	60.53	5′-CAAATCCTTTTGGTGGGAATC-3′	145
	Reverse	59.30	5′-CTATAGAAATTTCGCAAGAACTTAACC-3′	
Metalloprotease transcripts	Forward	60.5	5′-ATGAATGACGGTTCCCATGTA-3′	114
	Reverse	60.17	5′-GGCATTAAGGCAGCTCACTCT-3′	
FBox transcripts	Forward	60.25	5′-AGATAGGGAAATGTTGCAGGT-3′	93
	Reverse	59.84	5′-CTAATGGCAATTGCAGCTCTC-3′	

*Calculated Tm under PCR conditions.

### Silencing and overexpression vectors construction

**Figure 7:**
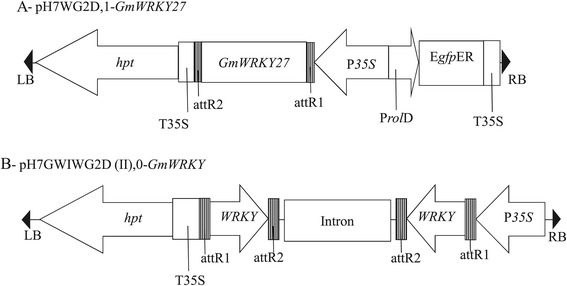
**T-DNA region of binary vectors used for** ***Gm*** **WRKY27 overexpression or** ***Gm*** **WRKY silence in soybean. (A)** Overexpression construct - pH7WG2D.1-*GmWRKY27*. The full-length ORF of *GmWRKY27* was cloned in the vector. **(B)** RNAi suppression construct - pH7GWIWG2(II).0-*GmWRKY*. Inverted repeats of a 176-bp WRKY fragment was cloned into the vector. RB – T-DNA right border, LB – left border, *hpt* – hygromycin phosphotransferase gene, P35S – Cauliflower mosaic virus (CaMV) 35S promoter, T35S – CaMV 35S terminator, EgfpER – enhanced green fluorescent protein, ProlD – root loci D promoter, *WRKY* – soybean 176 pb WRKY fragment, attB1 and attB2 – LR reaction site.

The open reading frame (ORF) of *GmWRKY27* (Glyma15g00570), according to Phytozome v1.0, was amplified from the MGBR-46 Conquista soybean cultivar using a high-fidelity Taq DNA Polymerase (*Pfu*–Fermentas). The Gateway® System (Invitrogen) was used to recombine the PCR product into the overexpression pH7WG2D,1 vector [91]. The T-DNA region of the resulting pH7WG2D,1-*GmWRKY27* vector contained the *GmWRKY27* gene ORF under the control of the CaMV *35S* promoter, the hygromycin-phosphotransferase marker gene (*hpt*) and the green fluorescent protein reporter gene (*gfp*) (Figure 7A). A RNAi silencing vector was constructed using pH7GWIWG2(II),0 [91]. The T-DNA region of the resulting pH7GWIWG2(II),0-*GmWRKY27* contained inverted repeat fragments (176 bp) from the *GmWRKY27* sequence, which were separated by an intron from the *Arabidopsis* genomic DNA sequence, under the control of the CaMV *35S* promoter and the hygromycin-phosphotransferase marker gene (*hpt*) (Figure 7B). Both constructs were confirmed using DNA sequencing.

### Soybean transformation and plant regeneration

Pods containing immature seeds of 3-5 mm in length from soybean cultivars MGBR 46 (Conquista), BRSMG 68 (Vencedora) and IAS5 were harvested from field grown plants. They are all susceptible to *P. pachyrhizi*. Somatic embryogenesis was induced from immature cotyledons and proliferated using the methods of Droste et al. [92]. Proliferating embryogenic tissues were subjected to transformation through particle bombardment using a particle inflow gun (PIG) [93] following the procedure of Droste et al. [92] or using the combined methods of DNA-free particle bombardment and *Agrobacterium* transformation [94]. After cultivating for three months in hygromycin-B selection medium, the hygromycin-resistant embryogenic soybean tissues were visually selected and individually cultured for the establishment of lines corresponding to putative independent transformation events.

Embryo histodifferentiation, conversion into plants and acclimation were carried out as previously described [92]. All plants derived from an independent sample of hygromycin-resistant tissue were considered as cloned plants. The plants derived from non-transformed embryogenic tissues submitted to the same culture conditions were recovered and used as controls for molecular characterization and bioassays.

### Screening for transgenic embryos and plants

Total DNA was extracted [95] from hygromycin-resistant histodifferentiated embryos and plant leaves. The putative transgenic embryos/plants were PCR-screened for the presence of the complete T-DNA using different primer combinations (Table 5). The PCR mixture consisted of 200 ng of template DNA, 0.4 mM of dNTPs, 0.4 μM of each primer, 2.5 mM of MgCl_2,_ 1X Taq Buffer, 1 U of Taq DNA Polymerase (Invitrogen), and autoclaved distilled water in a final volume of 25 μl. The reactions were initially heated (5 min at 94°C) and subjected to 30 cycles of the following conditions: 45 s at 94°C, 45 s at 58°C and 1 min at 72°C. Subsequent to electrophoresis on a 1% agarose gel containing ethidium bromide (0.01 mg/L), the PCR products were visualized under ultraviolet light.

**Table 5:** **Primer set designed to gene isolation and transgene detection**

**Target**	**Orientation**	**Tm (°C)**	**Primer Sequence**	**PCR product size (pb)**
35S	Forward	52	5′-GGACCCCCACCCACGAGGAG-3′	139*
*GmWRKY27* overexpression	Forward	58	5′- CACCATGGATTATTCATCATGGATTAACA-3′	921
	Reverse		5′- TTAATTATTATTGTGCAACATTTTTC-3′	
WRKY (RNAi)	Forward	58	5′- CACCCTTCCTTGGATCTCAACATTAATCT -3′	176
	Reverse		5′- TAACTTCTTGTTTTCTGCACTCACC -3′	
Intron	Forward	60	5′- TGCCTCTTCTTACGGCTTTCTGTG -3′	400
	Reverse		5′ - TGCCGTCTGTGATGGCTTCCA -3′	
Hpt	Forward	60	5′-GCGATTGCTGATCCCCATGTGTGTAT-3′	512
	Reverse		5′-GGTTTCCACTATCGGCGAGTACTT-3′	

*Fragment length was considered from the beginning of the primer sequence until the end of the promoter sequence.

GFP expression was detected under blue light using an Olympus® fluorescence stereomicroscope equipped with a BP filter set containing a 488 nm excitation filter and a 505-530 nm emission filter. The images were captured using the QCapture Pro™ 6 software (QImaging®).

Gene overexpression or silencing was confirmed using RT-qPCR. The RNA extraction, cDNA synthesis and qPCR analysis were performed as described above.

### Fungal bioassay

A detached leaf method was used to evaluate the *P. pachyrhizi* infection [49]. Three fully expanded leaves from each one transgenic line and two wild-type plants (2-month-old) were collected, rinsed with sterile distilled water and cut in 5 cm × 5 cm pieces. For the inoculation, 1 mL of a uredospore suspension (10^5^ spores/mL) was dripped onto each leaf piece, which was subsequently placed with its abaxial side upwards in a Petri dish covered with wet filter paper. The material was incubated at 20°C under a 12/12 h light/dark cycle. The number of lesions and pustules (uredium) was recorded at 12 days after inoculation. A non-parametric Student’s *t*-test was conducted to compare the effect of *P. pachyrhizi* on transgenic and non-transgenic plants. The results with p < 0.05 were considered significant.

## Additional files

Additional file 1:
**Annotation of** ***Glycine max*** **WRKY transcription factors (Chromosome 4 to 20).**
Additional file 2:
**Pseudogenes list.**
Additional file 3:
**Phylogenetic tree representing relationship among WRKY proteins of three species.** The tree was reconstructed using a Bayesian (BA) method. A total of 289 amino acid sequences from *Glycine max, Arabidopsis thaliana* and *Populus trichocarpa* and 65 sites corresponding to WRKY domain were included in the analysis. The posteriori probability values are labeled above the branches and only values higher than 70% are presented. The groups I, IIa, IIb, IIc, IId, IIe and III are indicated. *Differentially expressed genes in response to *P. pachyrhizi* infection. Additional file 4:
**Expression pattern of** ***WRKY*** **encoding-genes under** ***P. pachyrhizi*** **infection (Group IIb to III).**
Additional file 5:
**Characterization of soybean transgenic lines overexpressing** ***GmWRKY27*** **.** GFP expression analyses in wild type **(A)** and hygromycin-resistant embryogenic tissues **(B)**. GFP expression was detected under blue light using a fluorescence stereomicroscope Olympus®, equipped with a BP filter set containing a 488 nm excitation filter and a 505-530 nm emission filter. **(C)** Expression levels (RT-qPCR) of the *GmWRKY27* in wild-type (WT) soybean plants and in histodifferentiated embryos of different transgenic soybean lines. Venc (BRSMG68 Vencedora) P2-1, IAS-5 P1-1, Conq (MGBR-46 Conquista) P1-1 lines were obtained from Biobalistic and IAS-5 P3-1 line from Biobalistic/*Agrobacterium* transformation experiments. F-Box protein and metalloprotease reference genes were used as internal controls to normalize the amount of mRNA present in each sample. Transcript levels of WRKY genes present in the wt were used to calibrate the transcript amounts in transgenic embryos. *Means are significantly different in the wt and transgenic lines (Student’s *t*-test, p < 0.05).
