# Supplementary material for: Genome-wide annotation of the soybean WRKY family and functional characterization of genes involved in response to Phakopsora pachyrhizi infection
Source: BMC Plant Biol. 2014 Sep 10;14:236. doi: 10.1186/s12870-014-0236-0 (PMC4172953; doi:10.1186/s12870-014-0236-0)
Supplement: Additional file 1: — Annotation of Glycine max WRKY transcription factors (Chromosome 4 to 20). [file 12870_2014_236_MOESM1_ESM.docx]

| **Additional File 1: Annotation of Glycine max WRKY transcription factors (Chromosome 4 to 20)** | | | | | | | | | | | | | | | | | | |
| --- | --- | --- | --- | --- | --- | --- | --- | --- | --- | --- | --- | --- | --- | --- | --- | --- | --- | --- |
| **Chr** | | **Gene ID^a^** | **Name**^b^ | | **Alternative transcripts** | **CDS (pb)** | **Protein (aa)** | **Groups^c^** | **Expression** | **Soybase** | **Domain modifications** | | | | | | | |
|  |  | **(Phytozome)** |  |  |  |  |  |  | **confirmed^d^** | **EST ID** |  |  |  |  |  |  |  |  |
| 4 | | Glyma04g05700 | *GmWRKY79* | | 1 | 486 | 162 | IIc | + | FK004547.1 | WRKYGQK → WRKYG**K**K | | | | | | | |
| 4 | | Glyma04g06470 | *GmWRKY80* | | 1 | 669 | 223 | IIa | + | - |  | | | | | | | |
| 4 | | Glyma04g06495 | *GmWRKY81* | | 2 | 663 | 221 | IIa | + | EV275620.1 |  | | | | | | | |
| 4 | | Glyma04g08060 | *GmWRKY50* | | 1 | 840 | 280 | IId | + | EU019586.1 |  | | | | | | | |
| 4 | | Glyma04g12830 | *GmWRKY82* | | 2 | 2286 | 762 | I | + | BI471108.1 |  | | | | | | | |
| 4 | | Glyma04g34220 | *GmWRKY83* | | 1 | 1596 | 532 | IIb | + | - |  | | | | | | | |
| 4 | | Glyma04g39621 | *GmWRKY45* | | 2 | 705 | 235 | IIc | + | EU019581.1 |  | | | | | | | |
| 4 | | Glyma04g39650 | *GmWRKY21* | | 1 | 591 | 197 | IIc | + | DQ322691.1 | WRKYGQK → WRKYG**K**K | | | | | | | |
| 4 | | Glyma04g40121 | *GmWRKY84* | | 1 | 1014 | 338 | III | - | - |  | | | | | | | |
| 4 | | Glyma04g40130 | *GmWRKY58* | | 1 | 954 | 318 | III | + | EU375354.1 |  | | | | | | | |
| 4 | | Glyma04g41701 | *GmWRKY85* | | 1 | 1095 | 365 | III | + | BG653111.1 |  | | | | | | | |
| 5 | | Glyma05g01285 | *GmWRKY86* | | 2 | 1785 | 595 | IIb | + | GR858756.1 |  | | | | | | | |
| 5 | | Glyma05g20710 | *GmWRKY11* | | 2 | 1005 | 335 | IId | + | EU375356.1 |  | | | | | | | |
| 5 | | Glyma05g25270 | *GmWRKY87* | | 1 | 1086 | 362 | IIb | + | EV280519.1 | CX_(N)_CX_(N)_HXH/C → CX_(N)_CX_(N)_HX**T** | | | | | | | |
| 5 | | Glyma05g25331 | *GmWRKY88* | | 1 | 1293 | 431 | IIe | - | - |  | | | | | | | |
| 5 | | Glyma05g25770 | *GmWRKY89* | | 1 | 1077 | 359 | IIc | + | BW658787.1 |  | | | | | | | |
| 5 | | Glyma05g29310 | *GmWRKY90* | | 1 | 768 | 256 | IIe | + | GR846019.1 |  | | | | | | | |
| 5 | | Glyma05g29921 | *GmWRKY91* | | 2 | 4068 | 256 | III | - | - |  | | | | | | | |
| 5 | | Glyma05g31800 | *GmWRKY92* | | 1 | 567 | 189 | IIc | + | BT091158.1 | WRKYGQK → WRKYG**K**K | | | | | | | |
| 5 | | Glyma05g31910 | *GmWRKY93* | | 1 | 651 | 217 | IIc | - | - |  | | | | | | | |
| 5 | | Glyma05g36970 | *GmWRKY46* | | 1 | 1092 | 364 | III | + | EH258436.1 |  | | | | | | | |
| 5 | | Glyma05g37390 | *GmWRKY94* | | 1 | 867 | 289 | IIe | + | - |  | | | | | | | |
| 6 | | Glyma06g05721 | *GmWRKY95* | | 1 | 528 | 176 | IIc | - | - | WRKYGQK → WRKYG**K**K; CX_(N)_CX_(N)_HXH/C → CX_(N)_**W**X_(N)_HXH | | | | | | | |
| 6 | | Glyma06g06530 | *GmWRKY17* | | 1 | 885 | 295 | IIa | + | BT095976.1 |  | | | | | | | |
| 6 | | Glyma06g08120 | *GmWRKY37* | | 1 | 903 | 301 | IId | + | EU375346.1 |  | | | | | | | |
| 6 | | Glyma06g13090 | *GmWRKY96* | | 1 | 1095 | 365 | III | + | FK007605.1 |  | | | | | | | |
| 6 | | Glyma06g14720 | *GmWRKY97* | | 1 | 960 | 320 | III | + | AK244967.1 |  | | | | | | | |
| 6 | | Glyma06g14731 | *GmWRKY98* | | 2 | 996 | 332 | III | - | - |  | | | | | | | |
| 6 | | Glyma06g15220 | *GmWRKY61* | | 1 | 591 | 197 | IIc | + | EH220254.1 | WRKYGQK → WRKYG**K**K | | | | | | | |
| 6 | | Glyma06g15260 | *GmWRKY99* | | 2 | 711 | 237 | IIc | + | - |  | | | | | | | |
| 6 | | Glyma06g17690 | *GmWRKY100* | | 1 | 483 | 161 | IIc | - | - | WRKYGQK → WRKYG**K**K | | | | | | | |
| 6 | | Glyma06g20300 | *GmWRKY101* | | 1 | 1848 | 616 | IIb | + | DB968378.1 |  | | | | | | | |
| 6 | | Glyma06g23990 | *GmWRKY102* | | 1 | 729 | 243 | IIa | - | - | WRKYGQK → W**K**KYGQK | | | | | | | |
| 6 | | Glyma06g27440 | *GmWRKY103* | | 2 | 1413 | 471 | I | + | BT093246.1 |  | | | | | | | |
| 6 | | Glyma06g37100* | *GmWRKY104* | | 1 | 531 | 177 | I | - | - |  | | | | | | | |
|  | |  |  | |  |  |  |  |  |  | (Table continues on facing page) | | | | | | | |
| **Additional File 1. (Continued from previous page)** | | | | | | | | | | | | | | | | | | |
| **Chr** | | **Gene ID^a^** | | **Name**^b^ | **Alternative transcripts** | **CDS (pb)** | **Protein (aa)** | **Groups^c^** | **Expression** | **Soybase** | **Domain modifications** | | | | | | | |
|  |  | **(Phytozome)** | |  |  |  |  |  | **confirmed^d^** | **EST ID** |  |  |  |  |  |  |  |  |
| 6 | | Glyma06g46420 | | *GmWRKY105* | 1 | 1887 | 629 | IIb | + | CO979705.1 |  | | | | | | | |
| 6 | | Glyma06g47880 | | *GmWRKY59* | 1 | 2331 | 777 | I | + | CA801226.1 |  | | | | | | | |
| 7 | | Glyma07g02630 | | *GmWRKY106* | 1 | 936 | 312 | IIa | + | AK245986.1 |  | | | | | | | |
| 7 | | Glyma07g06320 | | *GmWRKY55* | 1 | 1110 | 370 | III | + | BT093956.1 |  | | | | | | | |
| 7 | | Glyma07g13611 | | *GmWRKY107* | 1 | 714 | 238 | IIc | - | - |  | | | | | | | |
| 7 | | Glyma07g16040 | | *GmWRKY108* | 1 | 954 | 318 | IIc | - | - |  | | | | | | | |
| 7 | | Glyma07g35381 | | *GmWRKY109* | 1 | 1602 | 534 | I | + | - |  | | | | | | | |
| 7 | | Glyma07g36640 | | *GmWRKY110* | 2 | 1176 | 392 | IIc | + | DB967901.1 |  | | | | | | | |
| 7 | | Glyma07g39250 | | *GmWRKY34* | 1 | 1731 | 577 | IIb | + | EU019573.1 |  | | | | | | | |
| 8 | | Glyma08g01430 | | *GmWRKY25* | 1 | 444 | 148 | IIc | + | EU019567.1 | WRKYGQK → WRKYG**E**K | | | | | | | |
| 8 | | Glyma08g02160 | | *GmWRKY111* | 1 | 879 | 293 | IIe | + | CA938468.1 |  | | | | | | | |
| 8 | | Glyma08g02580 | | *GmWRKY20* | 1 | 1080 | 360 | III | + | BT095747.1 |  | | | | | | | |
| 8 | | Glyma.08G078100 | | *GmWRKY112* | 1 | 546 | 182 | IIb | + | BI320613.1 |  | | | | | | | |
| 8 | | Glyma08g08340 | | *GmWRKY113* | 1 | 1290 | 430 | IIe | + | - |  | | | | | | | |
| 8 | | Glyma08g08720 | | *GmWRKY114* | 1 | 1116 | 372 | IIc | + | - |  | | | | | | | |
| 8 | | Glyma08g12460 | | *GmWRKY48* | 1 | 786 | 262 | IIe | + | GR844807.1 |  | | | | | | | |
| 8 | | Glyma08g15050 | | *GmWRKY6* | 1 | 555 | 185 | IIc | + | EH260054.1 | WRKYGQK →WRKYG**K**K | | | | | | | |
| 8 | | Glyma08g15210 | | *GmWRKY40* | 3 | 708 | 236 | IIc | + | DQ322692.1 |  | | | | | | | |
| 8 | | Glyma08g23380 | | *GmWRKY56* | 4 | 942 | 314 | IIa | + | EU375348.1 |  | | | | | | | |
| 8 | | Glyma08g26230 | | *GmWRKY4* | 1 | 1572 | 524 | I | + | EU375355.1 |  | | | | | | | |
| 8 | | Glyma08g43258 | | *GmWRKY115* | 1 | 1461 | 487 | IIb | + | - |  | | | | | | | |
| 8 | | Glyma08g43770 | | *GmWRKY18* | 1 | 1734 | 578 | I | + | BU080760.1 |  | | | | | | | |
| 9 | | Glyma09g00820 | | *GmWRKY23* | 1 | 1626 | 542 | IIb | + | AK245933.1 |  | | | | | | | |
| 9 | | Glyma09g03451 | | *GmWRKY116* | 2 | 1521 | 507 | IIe | - | - |  | | | | | | | |
| 9 | | Glyma09g03900 | | *GmWRKY26* | 1 | 996 | 332 | IIc | + | GR833156.1 |  | | | | | | | |
| 9 | | Glyma09g06980 | | *GmWRKY47* | 1 | 891 | 297 | IId | + | EU019583.1 |  | | | | | | | |
| 9 | | Glyma09g09400 | | *GmWRKY117* | 1 | 1377 | 459 | IIb | + | CF808085.1 |  | | | | | | | |
| 9 | | Glyma.09g127100 | | *GmWRKY118* | 1 | 729 | 242 | IIb | - | - | CX(N)CX(N)HXH/C → CX(N)CX(N) | | | | | | | |
| 9 | | Glyma09g24080 | | *GmWRKY119* | 1 | 1119 | 373 | IIe | + | - |  | | | | | | | |
| 9 | | Glyma09g37470 | | *GmWRKY120* | 1 | 1626 | 542 | IIb | + | - |  | | | | | | | |
| 9 | | Glyma09g37930 | | *GmWRKY121* | 3 | 717 | 239 | IIc | + | BT091680.1 |  | | | | | | | |
| 9 | | Glyma09g38581 | | *GmWRKY122* | 1 | 2205 | 735 | I | + | - |  | | | | | | | |
| 9 | | Glyma09g39000 | | *GmWRKY123* | 1 | 579 | 193 | IIc | + | AW307474.1 |  | | | | | | | |
| 9 | | Glyma09g39040 | | *GmWRKY124* | 1 | 1047 | 349 | IIe | + | BW658775.1 |  | | | | | | | |
| 9 | | Glyma09g41050 | | *GmWRKY125* | 1 | 903 | 301 | III | + | BT098696.1 |  | | | | | | | |
|  | |  | |  |  |  |  |  |  |  | (Table continues on facing page) | | | | | | | |
| **Additional File 1. (Continued from previous page)** | | | | | | | | | | | | | | | | | | |
| **Chr** | | **Gene ID^a^** | | **Name**^b^ | **Alternative transcripts** | **CDS (pb)** | **Protein (aa)** | **Groups^c^** | **Expression** | **Soybase** | **Domain modifications** | | | | | | | |
|  |  | **(Phytozome)** | |  |  |  |  |  | **confirmed^d^** | **EST ID** |  |  |  |  |  |  |  |  |
| 9 | | Glyma09g41670 | | *GmWRKY126* | 2 | 1632 | 544 | I | + | - | WRKYGQK →W**I**KYGQK (N-terminal) | | | | | | | |
| 10 | | Glyma10g01450 | | *GmWRKY54* | 2 | 972 | 324 | IIc | + | DQ322698.1 |  | | | | | | | |
| 10 | | Glyma10g03820 | | *GmWRKY127* | 2 | 1179 | 393 | IId | + | DB971123.1 |  | | | | | | | |
| 10 | | Glyma10g13720 | | *GmWRKY128* | 1 | 363 | 121 | IIa | + | CO980573.1 | WRKYGQK → WRKYG**K**K; CX_(N)_CX_(N)_HXH/C → CX_(N)_CX_(N)_  (C-terminal) | | | | | | | |
| 10 | | Glyma10g14610 | | *GmWRKY129* | 1 | 957 | 319 | IIb | - | - | WRKYGQK → **C**RKYGQK; CX_(N)_CX_(N)_HXH/C → CX_(N)_CX_(N)_HX**N** | | | | | | | |
| 10 | | Glyma10g27860 | | *GmWRKY1* | 4 | 1449 | 483 | IIb | + | BI426286.1 |  | | | | | | | |
| 10 | | Glyma10g31396 | | *GmWRKY130* | 1 | 579 | 193 | IIc | - | - | WRKYGQK→W**HQ**YG**L**K | | | | | | | |
| 10 | | Glyma10g31413 | | *GmWRKY131* | 1 | 1011 | 337 | IIc | - | - |  | | | | | | | |
| 10 | | Glyma10g37460 | | *GmWRKY2* | 1 | 894 | 298 | IIe | + | CO980938.1 |  | | | | | | | |
| 11 | | Glyma11g02361 | | *GmWRKY132* | 1 | 645 | 215 | IIe | + | - | CX_(N)_CX_(N)_HXH/C → CX_(N)_CX_(N)_HX**D** | | | | | | | |
| 11 | | Glyma11g05650 | | *GmWRKY14* | 2 | 966 | 322 | IId | + | EU019559.1 |  | | | | | | | |
| 11 | | Glyma11g29720 | | *GmWRKY19* | 1 | 1647 | 549 | I | + | AK245549.1 |  | | | | | | | |
| 12 | | Glyma12g10350 | | *GmWRKY133* | 1 | 1845 | 615 | IIb | + | BG839187.1 |  | | | | | | | |
| 12 | | Glyma12g23950 | | *GmWRKY44* | 2 | 1404 | 468 | I | + | [EV282360.1](http://www.ncbi.nlm.nih.gov/nucleotide/151934204?report=genbank&log$=nucltop&blast_rank=2&RID=C4Z75BP501N) |  | | | | | | | |
| 12 | | Glyma12g33990 | | *GmWRKY16* | 1 | 792 | 264 | IIe | + | EU375351.1 |  | | | | | | | |
| 13 | | Glyma13g00380 | | *GmWRKY13* | 1 | 975 | 325 | IId | + | DQ322694.1 |  | | | | | | | |
| 13 | | Glyma13g17800 | | *GmWRKY134* | 1 | 1152 | 384 | IIb | + | CF807620.1 |  | | | | | | | |
| 13 | | Glyma13g34240 | | *GmWRKY135* | 1 | 885 | 295 | III | - | - |  | | | | | | | |
| 13 | | Glyma13g34251 | | *GmWRKY136* | 1 | 891 | 297 | III | - | - |  | | | | | | | |
| 13 | | Glyma13g34261 | | *GmWRKY137* | 1 | 903 | 301 | III | - | - |  | | | | | | | |
| 13 | | Glyma13g34281 | | *GmWRKY138* | 1 | 813 | 271 | III | - | - |  | | | | | | | |
| 13 | | Glyma13g36540 | | *GmWRKY52* | 1 | 798 | 266 | IIe | + | CF807263.1 |  | | | | | | | |
| 13 | | Glyma13g38630 | | *GmWRKY36* | 1 | 1845 | 615 | IIb | + | EU019575.1 |  | | | | | | | |
| 13 | | Glyma13g44730 | | *GmWRKY139* | 1 | 930 | 310 | IIa | + | AK244287.1 |  | | | | | | | |
| 14 | | Glyma14g01010 | | *GmWRKY140* | 2 | 1527 | 509 | I | + | BM523517.1 |  | | | | | | | |
| 14 | | Glyma14g01980 | | *GmWRKY7* | 1 | 1758 | 586 | I | + | EH257685 |  | | | | | | | |
| 14 | | Glyma14g03280 | | *GmWRKY141* | 1 | 1008 | 336 | IIc | + | FK018954.1 |  | | | | | | | |
| 14 | | Glyma14g11440 | | *GmWRKY142* | 1 | 414 | 138 | IIc | - | - | WRKYGQK → WRKYG**K**K | | | | | | | |
| 14 | | Glyma14g11920 | | *GmWRKY143* | 1 | 837 | 279 | IIa | + | CD410097.1 |  | | | | | | | |
| 14 | | Glyma14g11960 | | *GmWRKY144* | 1 | 849 | 283 | IIa | + | BF009368.1 |  | | | | | | | |
| 14 | | Glyma14g17730 | | *GmWRKY31* | 1 | 951 | 317 | IId | + | GR845636.1 |  | | | | | | | |
| 14 | | Glyma14g36430 | | *GmWRKY145* | 1 | 990 | 330 | III | + | EV266531.1 |  | | | | | | | |
| 14 | | Glyma14g36438 | | *GmWRKY146* | 2 | 912 | 304 | III | + | AI966709.1 |  | | | | | | | |
| 14 | | Glyma14g36446 | | *GmWRKY147* | 1 | 723 | 241 | III | - | - |  | | | | | | | |
| 14 | | Glyma14g37960 | | *GmWRKY148* | 1 | 999 | 333 | IIc | + | FG986745.1 | WRKYGQK → WRKY**ED**K | | | | | | | |
|  | |  | |  |  |  |  |  |  |  | (Table continues on facing page) | | | | | | | |
| **Additional File 1. (Continued from previous page)** | | | | | | | | | | | | | | | | | | |
| **Chr** | | **Gene ID^a^** | | **Name**^b^ | **Alternative transcripts** | **CDS (pb)** | **Protein (aa)** | **Groups^c^** | **Expression** | **Soybase** | **Domain modifications** | | | | | | | |
|  |  | **(Phytozome)** | |  |  |  |  |  | **confirmed^d^** | **EST ID** |  |  |  |  |  |  |  |  |
| 14 | | Glyma14g38010 | | *GmWRKY49* | 2 | 1728 | 576 | I | + | EU019585.1 |  | | | | | | | |
| 15 | | Glyma15g00570 | | *GmWRKY27* | 1 | 993 | 331 | IIa | + | DQ322695.1 |  | | | | | | | |
| 15 | | Glyma15g11680 | | *GmWRKY149* | 1 | 1800 | 600 | IIb | + | GR856238.1 |  | | | | | | | |
| 15 | | Glyma15g14371 | | *GmWRKY150* | 2 | 1572 | 524 | IIe | - | - |  | | | | | | | |
| 15 | | Glyma15g14860 | | *GmWRKY151* | 2 | 1071 | 357 | IIc | + | BW658350.1 |  | | | | | | | |
| 15 | | Glyma15g18250 | | *GmWRKY42* | 1 | 882 | 294 | IId | + | EU019578.1 |  | | | | | | | |
| 15 | | Glyma15g20990 | | *GmWRKY152* | 1 | 1356 | 452 | IIb | + | BW662875.1 |  | | | | | | | |
| 16 | | Glyma16g02960 | | *GmWRKY60* | 1 | 1122 | 374 | III | + | BT095906.1 |  | | | | | | | |
| 16 | | Glyma16g03480 | | *GmWRKY153* | 2 | 588 | 196 | IIc | + | BG650157.1 |  | | | | | | | |
| 16 | | Glyma16g03570 | | *GmWRKY154* | 1 | 1008 | 336 | IIe | + | FG989867.1 |  | | | | | | | |
| 16 | | Glyma16g05880 | | *GmWRKY155* | 1 | 588 | 196 | IIc | + | GR835181.1 |  | | | | | | | |
| 16 | | Glyma.16G176700 | | *GmWRKY156* | 1 | 825 | 275 | IIe | - | - |  | | | | | | | |
| 16 | | Glyma16g29561 | | *GmWRKY157* | 1 | 1227 | 409 | IIe | + | EH260684.1 |  | | | | | | | |
| 16 | | Glyma16g34590 | | *GmWRKY158* | 1 | 798 | 266 | III | + | - |  | | | | | | | |
| 17 | | Glyma17g01490 | | *GmWRKY159* | 2 | 1470 | 490 | IIb | + | BE057555.1 |  | | | | | | | |
| 17 | | Glyma17g03950 | | *GmWRKY160* | 2 | 1197 | 399 | IIc | + | GR855044.1 |  | | | | | | | |
| 17 | | Glyma17g04710 | | *GmWRKY161* | 1 | 1176 | 392 | IIb | + | BG726970.1 |  | | | | | | | |
| 17 | | Glyma17g06450 | | *GmWRKY33* | 1 | 963 | 321 | IId | + | DQ322696.1 |  | | | | | | | |
| 17 | | Glyma17g08170 | | *GmWRKY24* | 1 | 1518 | 506 | I | + | AK245832.1 |  | | | | | | | |
| 17 | | Glyma17g10630 | | *GmWRKY162* | 1 | 1803 | 601 | IIb | + | - |  | | | | | | | |
| 17 | | Glyma17g18480 | | *GmWRKY163* | 1 | 999 | 333 | IId | + | BE473968.1 |  | | | | | | | |
| 17 | | Glyma17g29190 | | *GmWRKY164* | 1 | 951 | 317 | IId | + | CK768919.1 |  | | | | | | | |
| 17 | | Glyma17g33891 | | *GmWRKY30* | 1 | 939 | 313 | IIa | + | EU019570.1 | CX_(N)_CX_(N)_HXH/C → CX_(N)_CX_(N)_ | | | | | | | |
| 17 | | Glyma17g33920 | | *GmWRKY63* | 1 | 837 | 279 | IIa | + | BT095938.1 |  | | | | | | | |
| 17 | | Glyma17g34210 | | *GmWRKY165* | 2 | 495 | 165 | IIc | + | - | WRKYGQK →WRKYG**K**K | | | | | | | |
| 17 | | Glyma17g35750 | | *GmWRKY166* | 1 | 837 | 279 | IId | + | - | CX_(N)_CX_(N)_HXH/C → - | | | | | | | |
| 18 | | Glyma18g06360 | | *GmWRKY62* | 1 | 1629 | 543 | I | + | AK245515.1 |  | | | | | | | |
| 18 | | Glyma18g09040 | | *GmWRKY167* | 3 | 1734 | 578 | I | + | GD757522.1 |  | | | | | | | |
| 18 | | Glyma18g10324 | | *GmWRKY168* | 2 | 1437 | 479 | IIb | - | - |  | | | | | | | |
| 18 | | Glyma18g16170 | | *GmWRKY169* | 1 | 1590 | 530 | IIb | + | - |  | | | | | | | |
| 18 | | Glyma18g39970 | | *GmWRKY170* | 1 | 927 | 309 | IIc | + | - |  | | | | | | | |
| 18 | | Glyma18g44030 | | *GmWRKY171* | 2 | 1626 | 542 | I | + | BU764562.1 |  | | | | | | | |
| 18 | | Glyma18g44560 | | *GmWRKY57* | 1 | 900 | 300 | III | + | EU375353.1 |  | | | | | | | |
| 18 | | Glyma18g47300 | | *GmWRKY10* | 1 | 1056 | 352 | IIe | + | EU375344.1 |  | | | | | | | |
| 18 | | Glyma18g47350 | | *GmWRKY64* | 1 | 579 | 193 | IIc | + | EU019592.1 |  | | | | | | | |
|  | |  | |  |  |  |  |  |  |  | (Table continues on facing page) | | | | | | | |
| **Additional File 1. (Continued from previous page)** | | | | | | | | | | | | | | | | | | |
| **Chr** | | **Gene ID^a^** | | **Name**^b^ | **Alternative transcripts** | **CDS (pb)** | **Protein (aa)** | **Groups^c^** | **Expression** | **Soybase** | **Domain modifications** | | | | | | | |
|  |  | **(Phytozome)** | |  |  |  |  |  | **confirmed^d^** | **EST ID** |  |  |  |  |  |  |  |  |
| 18 | | Glyma18g47741 | | *GmWRKY172* | 1 | 2235 | 745 | I | - | - |  | | | | | | | |
| 18 | | Glyma18g48460 | | *GmWRKY38* | 1 | 678 | 225 | IIc | + | EU375347.1 |  | | | | | | | |
| 18 | | Glyma18g49140 | | *GmWRKY173* | 1 | 1626 | 542 | IIb | + | BU547639.1 |  | | | | | | | |
| 18 | | Glyma18g49830 | | *GmWRKY174* | 1 | 1563 | 521 | I | + | BQ742680.1 |  | | | | | | | |
| 19 | | Glyma19g02440 | | *GmWRKY175* | 1 | 1488 | 496 | IIb | + | BE020267.1 |  | | | | | | | |
| 19 | | Glyma19g26400 | | *GmWRKY53* | 1 | 567 | 189 | IIc | + | FK012118.1 |  | | | | | | | |
| 19 | | Glyma19g36100 | | *GmWRKY176* | 1 | 1416 | 472 | I | + | FK302159.1 |  | | | | | | | |
| 19 | | Glyma19g40470 | | *GmWRKY8* | 1 | 795 | 265 | IIe | + | EU019556.1 |  | | | | | | | |
| 19 | | Glyma19g40560 | | *GmWRKY177* | 1 | 873 | 291 | IIc | + | BI893911.1 |  | | | | | | | |
| 19 | | Glyma19g40950 | | *GmWRKY178* | 2 | 1551 | 517 | IIb | + | GR836108.1 |  | | | | | | | |
| 19 | | Glyma19g44380 | | *GmWRKY179* | 1 | 1089 | 363 | III | + | AW311275.1 |  | | | | | | | |
| 20 | | Glyma20g03410 | | *GmWRKY180* | 1 | 1320 | 440 | I | + | GR851585.1 |  | | | | | | | |
| 20 | | Glyma20g03820 | | *GmWRKY181* | 1 | 492 | 163 | IIb | - | - |  | | | | | | | |
| 20 | | Glyma20g30290 | | *GmWRKY182* | 1 | 966 | 322 | IIe | + | - |  | | | | | | | |
| ^a^ Reannotated genes with original sequences containing wrong start\stop codons are marked with (*).  ^b^The names *Gm*WRKY1-64 are given according to Zhou et al [38]; *Gm*WRKY65-182 are given according to the chromosome order | | | | | | | | | | | | | | | |  |  | |
| ^c^The classification according to Eugelm et al. [24] | | | | | | | | | | |  |  |  |  |  |  |  | |
| ^d^The expression confirmation according to Soybase ESTs and RNA-Seq analysis (in silico analysis) and RNA-Seq of ASR lesion microdissection (experimental analysis) | | | | | | | | | | | | | | | | | | |
