# Supplementary material for: Genome-wide annotation of the soybean WRKY family and functional characterization of genes involved in response to Phakopsora pachyrhizi infection
Source: BMC Plant Biol. 2014 Sep 10;14:236. doi: 10.1186/s12870-014-0236-0 (PMC4172953; doi:10.1186/s12870-014-0236-0)
Supplement: Additional file 2: — Pseudogenes list. [file 12870_2014_236_MOESM2_ESM.docx]

| Additional file 2. Pseudogenes list | |  |  |  | |
| --- | --- | --- | --- | --- | --- |
| **Pseudogene name*** | **Gene Model (Phytozome)** | **Location*** | **Comments** | **USM Comments** | |
| GmWRKYpg01 | - | Gm01:43400758..43405757 | - | In frame stops. Next to transposon-like sequence | |
| GmWRKYpg02 | Glyma12g29970 | Gm12:33436284..33441283 | Incomplete domain | Missing C-terminal part. In frame stop and GAG poly. | |
| GmWRKYpg03 | - | Gm06:28589287..28599286 | - | In frame stops. Next to transposon-like sequence | |
| GmWRKYpg04 | - | Gm14:45750065..45770064 | - | WRKY missing, GAG poly. Next to WRKY170, 171,172 | |
| GmWRKYpg05 | - | Gm05:22316949..22326948 | - | WRKY missing, GAG poly. | |
| GmWRKYpg06 | - | Gm15:18509437..18519436 | - | WRKY missing, CACTA transposon. | |
| GmWRKYpg07 | - | Gm20:44350390..44360389 | - | C-terminal part of domain missing. No transposon. | |
| GmWRKYpg08 | - | Gm15:46738419..46748418 | - | C-terminal part of domain missing. Transposon. | |
| GmWRKYpg09 | - | Gm01:6267916,6277915 | - | No WRKY part | |
| GmWRKYpg10 | Glyma08g08370 | Gm08:6005538,6015537 | No WRKY domain | No WRKY part | |
| GmWRKYpg11 | - | Gm17:26744603,26754602 | - | No WRKY part | |
| GmWRKYpg12 | Glyma05g38200 | Gm05:41581323,41591322 | No WRKYGQK domain signature | No WRKY part. In frame stop. | |
| GmWRKYpg13 | - | Gm19:34108703,34113702 | - | C-terminal part of domain missing. Transposon. | |
| GmWRKYpg14 | - | Gm05:16280053,16290052 | - | WRKY missing. Transposon. | |
| GmWRKYpg15 | - | Gm16:24249352,24259351 | - | No WRKY part | |
| GmWRKYpg16 | Glyma12g31860 | Gm12:35418204..35419106 | Incomplete domain | No WRKY part | |
| GmWRKYpg18 | Glyma06g41910 | Gm06:45172914..45174125 | Truncated-5´; domain incomplete | In frame stops in WRKY | |
| GmWRKYpg19 | Glyma12g20900 | Gm12:22393464..22393609 | Incomplete domain | WRKY missing. Transposon. | |
| GmWRKYpg21 | - | Gm09:14588320,14589319 | - | WRKY missing. Transposon. | |
| GmWRKYpg22 | - | Gm20:32888766,32890765 | - | No WRKY part | |
| GmWRKYpg23 | Glyma17g24700 | Gm17:25313159..25313933 | Truncated-5´ | One domain missing large parts | |
| GmWRKYpg24 | - | Gm19:33166744,33171743 | - | WRKY missing. Transposon. | |
| GmWRKYpg25 | Glyma07g20510 | Gm07:20918593..20919466 | Truncated-5´; domain incomplete | In frame stop in WRKY. Transposon. | |
| GmWRKYpg26 | Glyma08g32740 | Gm08:28989837..28990680 | Incomplete domain | Gag-protease-integrase-RT-RNaseH downstream | |
|  |  |  |  | (Table continues on facing page) | |
| **Additional File 3. (Continued from previous page)** | | | | | |
| **Pseudogene name*** | **Gene Model (Phytozome)** | **Location*** | **Comments** | | **USM Comments** |
| GmWRKYpg27 | Glyma14g35150 | Gm14:43937946,43942945 | Incomplete domain | | WRKY missing. Transposon. |
| GmWRKYpg28 | - | Gm15:32213942,32223941 | - | | C-terminal part of domain missing. In frame stops. |
| GmWRKYpg29 | Glyma20g16010 | Gm20:22047658..22048200 | Incomplete domain | | Probable pseudogene as GYN*QKYK for GYNWRKY in second domain |
| GmWRKYpg30 | Glyma14g12290 | Gm14:11002636..11004011 | Truncated-3´; atypical WRKY signature (CRKYGQK) | | First domain in frame stop and frame shift |
| GmWRKYpg31 | - | Gm03:32340121,32360120 | - | | Possible pseudogene. Missing N-terminal part |
| GmWRKYpg33 | Glyma15g21570 | Gm15:19903373..19903444 | Incomplete domain | | Incomplete WRKY domain. |
| GmWRKYpg34 | Glyma15g37120 | Gm15:42731483..42732790 | Truncated-5´ | | Flanked by CACTA transposon sequences |
| GmWRKYpg35 | Glyma13g05720 | Gm13:6074457..6074710 | No WRKY domain signature | | No WRKY part |
| GmWRKYpg36 | Glyma17g25140 | Gm17:25787689..25788628 | Both domains seem non functional | | - |
| * Acording to USM database (http://systemsbiology.usm.edu/BrachyWRKY/WRKY/Soybean.html) | | | | |  |
