# Supplementary figures and images for: Genome-wide annotation of the soybean WRKY family and functional characterization of genes involved in response to Phakopsora pachyrhizi infection
Source: BMC Plant Biol. 2014 Sep 10;14:236. doi: 10.1186/s12870-014-0236-0 (PMC4172953; doi:10.1186/s12870-014-0236-0)

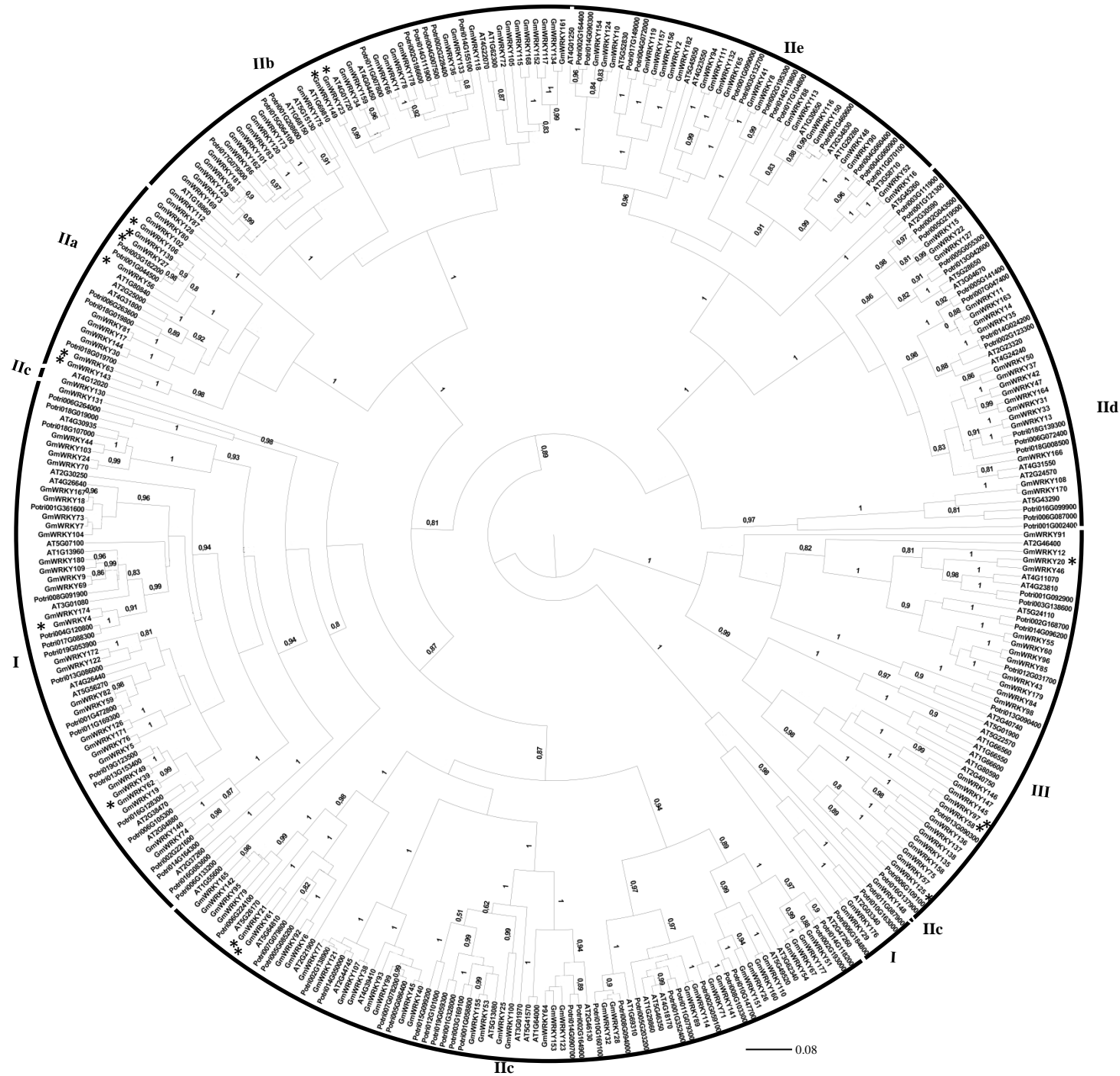

Supplement: Additional file 3: — Phylogenetic tree representing relationship among WRKY proteins of three species. The tree was reconstructed using a Bayesian (BA) method. A total of 289 amino acid sequences from Glycine max, Arabidopsis thaliana and Populus trichocarpa and 65 sites corresponding to WRKY domain were included in the analysis. The posteriori probability values are labeled above the branches and only values higher than 70% are presented. The groups I, IIa, IIb, IIc, IId, IIe and III are indicated. *Differentially expressed genes in response to P. pachyrhizi infection. [file 12870_2014_236_MOESM3_ESM.pdf]

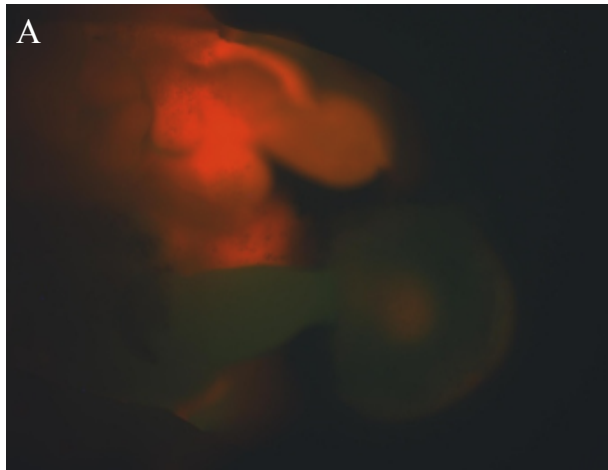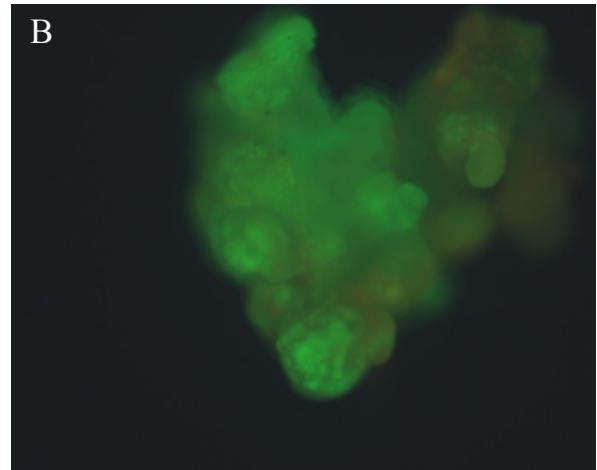

**C**

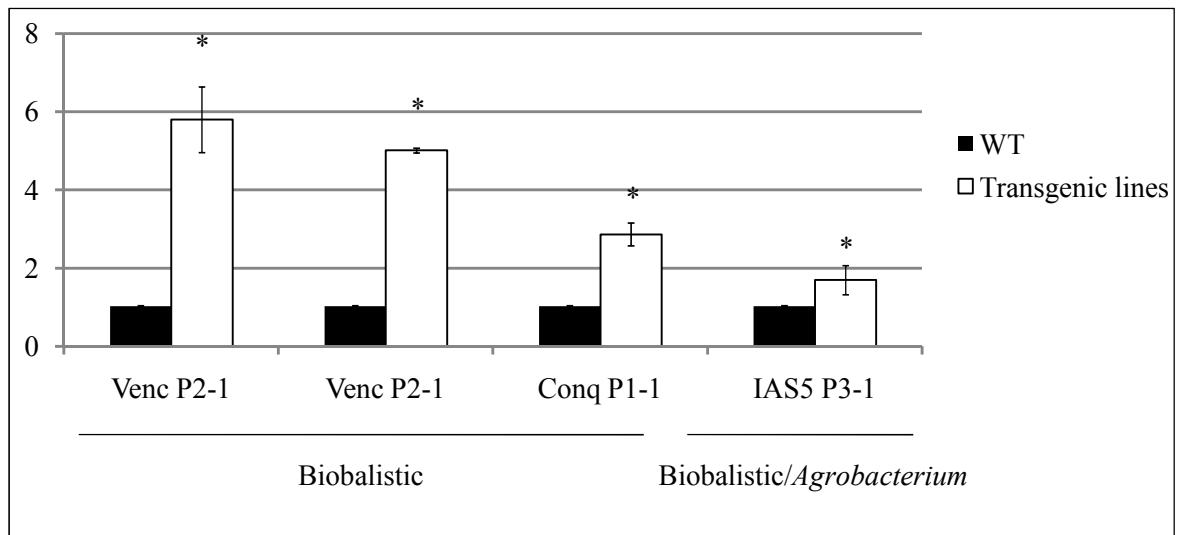

Supplement: Additional file 5: — Characterization of soybean transgenic lines overexpressing GmWRKY27 . GFP expression analyses in wild type (A) and hygromycin-resistant embryogenic tissues (B). GFP expression was detected under blue light using a fluorescence stereomicroscope Olympus®, equipped with a BP filter set containing a 488 nm excitation filter and a 505-530 nm emission filter. (C) Expression levels (RT-qPCR) of the GmWRKY27 in wild-type (WT) soybean plants and in histodifferentiated embryos of different transgenic soybean lines. Venc (BRSMG68 Vencedora) P2-1, IAS-5 P1-1, Conq (MGBR-46 Conquista) P1-1 lines were obtained from Biobalistic and IAS-5 P3-1 line from Biobalistic/Agrobacterium transformation experiments. F-Box protein and metalloprotease reference genes were used as internal controls to normalize the amount of mRNA present in each sample. Transcript levels of WRKY genes present in the wt were used to calibrate the transcript amounts in transgenic embryos. *Means are significantly different in the wt and transgenic lines (Student’s t-test, p < 0.05). [file 12870_2014_236_MOESM5_ESM.pdf]
