# Supplementary material for: Genome-wide annotation of the soybean WRKY family and functional characterization of genes involved in response to Phakopsora pachyrhizi infection
Source: BMC Plant Biol. 2014 Sep 10;14:236. doi: 10.1186/s12870-014-0236-0 (PMC4172953; doi:10.1186/s12870-014-0236-0)
Supplement: Additional file 4: — Expression pattern of WRKY encoding-genes under P. pachyrhizi infection (Group IIb to III). [file 12870_2014_236_MOESM4_ESM.docx]

| **Additional file 4. Expression pattern of WRKY encoding-genes under *P. pachyrhizi* infection^a^.** | | | | | | | | | | | | | | | | | | | | | | | | | | | | | | | | | | |  |
| --- | --- | --- | --- | --- | --- | --- | --- | --- | --- | --- | --- | --- | --- | --- | --- | --- | --- | --- | --- | --- | --- | --- | --- | --- | --- | --- | --- | --- | --- | --- | --- | --- | --- | --- | --- |
| **Group** | **Gene ID** | **SuperSage - LGE** | |  | **RNA-Seq of lesion LCM^b^** | | |  | | **Microarray - Mortel et al. [17]^c^** | | | | | | | | | |  | | **Microarray - Schneider et al. [22]** | | | | | | | | | | | | |  |
|  |  | **Incompatible reaction (PI561356-*Rpp1*)** | |  | **PI561356 X BRS231** | | |  | | **Incompatible reaction (PI230970-*Rpp2*)** | | | |  | | **Compatible reaction (Embrapa48)** | | | |  | | **Compatible reaction (PI462312-*Rpp3* X Taiwan 80-2)** | | | | | |  | | | **Incompatible reaction (PI462312-*Rpp3* X Hawaii 94-1)** | | | |  |
|  |  | **Inoculated X Mock** | |  | **Inoculated** | | |  | | **Inoculated X Mock** | | | |  | | **Inoculated X Mock** | | | |  | | **Inoculated X Mock** | | | | | |  | | | **Inoculated X Mock** | | | |  |
|  |  | **12, 24, 48h** | |  | **10 days** | | |  | | **12h** | | **120 h** | |  | | **12h** | | **120 h** | |  | | **12h** | | | **144h** | | |  | | | **12h** | | **144h** | |  |
| IIb | Glyma17g10630 |  | |  | x | | |  | |  | |  | |  | |  | |  | |  | |  | | |  | | |  | | |  | |  | |  |
| IIb | Glyma01g05050 |  | |  | x | | |  | |  | |  | |  | |  | |  | |  | |  | | |  | | |  | | |  | |  | |  |
| IIb | Glyma17g04710 |  | |  | x | | |  | |  | |  | |  | |  | |  | |  | |  | | |  | | |  | | |  | |  | |  |
| IIb | Glyma09g09400 |  | |  | x | | |  | |  | |  | |  | |  | |  | |  | |  | | |  | | |  | | |  | |  | |  |
| IIb | Glyma15g20990 |  | |  |  | | |  | |  | |  | |  | |  | |  | |  | |  | | | x | | |  | | |  | |  | |  |
| IIb | Glyma13g38630 |  | |  |  | | |  | |  | |  | |  | |  | |  | |  | | x | | | x | | |  | | | x | | x | |  |
| IIb | Glyma09g00820 | x | |  |  | | |  | | x | | x | |  | | x | | x | |  | |  | | |  | | |  | | |  | |  | |  |
| IIb | Glyma15g11680 |  | |  |  | | |  | | x | | x | |  | | x | | x | |  | | x | | | x | | |  | | | x | | x | |  |
| IIb | Glyma07g39250 |  | |  |  | | |  | |  | |  | |  | |  | |  | |  | | x | | |  | | |  | | | x | | x | |  |
| IIb | Glyma08g43260 |  | |  | x | | |  | |  | |  | |  | |  | |  | |  | |  | | |  | | |  | | |  | |  | |  |
| IIb | Glyma20g03820 | x | |  | x | | |  | |  | |  | |  | |  | |  | |  | |  | | |  | | |  | | |  | |  | |  |
| IIc | Glyma01g06870 |  | |  |  | | |  | |  | |  | |  | |  | |  | |  | |  | | |  | | |  | | | x | | x | |  |
| IIc | Glyma17g03950 |  | |  | x | | |  | |  | |  | |  | |  | |  | |  | |  | | |  | | |  | | |  | |  | |  |
| IIc | Glyma15g14860 |  | |  | x | | |  | |  | |  | |  | |  | |  | |  | |  | | |  | | |  | | |  | |  | |  |
| IIc | Glyma08g15050 |  | |  |  | | |  | | x | |  | |  | |  | | x | |  | |  | | |  | | |  | | |  | |  | |  |
| IIc | Glyma06g15220 | x | |  | x | | |  | | x | | x | |  | | x | | x | |  | |  | | |  | | |  | | |  | |  | |  |
| IIc | Glyma04g39650 |  | |  | x | | |  | | x | | x | |  | | x | | x | |  | |  | | |  | | |  | | |  | |  | |  |
| IIc | Glyma17g34210 |  | |  | x | | |  | |  | |  | |  | |  | |  | |  | |  | | |  | | |  | | |  | |  | |  |
| IIc | Glyma06g15260 |  | |  | x | | |  | |  | |  | |  | |  | |  | |  | |  | | |  | | |  | | |  | |  | |  |
| IIc | Glyma03g25770 |  | |  | x | | |  | |  | |  | |  | |  | |  | |  | |  | | |  | | |  | | |  | |  | |  |
| IIc | Glyma09g37930 |  | |  | x | | |  | |  | |  | |  | |  | |  | |  | |  | | |  | | |  | | |  | |  | |  |
| IIc | Glyma08g01430 |  | |  |  | | |  | | x | | x | |  | | x | | x | |  | |  | | |  | | |  | | |  | |  | |  |
| IIc | Glyma19g26400 |  | |  |  | | |  | | x | | x | |  | | x | | x | |  | |  | | |  | | |  | | |  | |  | |  |
| IId | Glyma06g08120 |  | |  |  | | |  | |  | |  | |  | |  | |  | |  | | x | | | x | | |  | | |  | |  | |  |
| IId | Glyma04g08060 |  | |  | x | | |  | |  | |  | |  | |  | |  | |  | |  | | |  | | |  | | |  | |  | |  |
| IId | Glyma17g29190 |  | |  |  | | |  | |  | |  | |  | |  | |  | |  | | x | | |  | | |  | | |  | | x | |  |
| IId | Glyma13g00380 |  | |  |  | | |  | |  | |  | |  | |  | |  | |  | | x | | |  | | |  | | | x | | x | |  |
| IId | Glyma17g06450 | x | |  |  | | |  | |  | |  | |  | |  | |  | |  | |  | | |  | | |  | | |  | |  | |  |
| IId | Glyma15g18250 |  | |  |  | | |  | | x | |  | |  | | x | |  | |  | |  | | |  | | |  | | |  | |  | |  |
| **(Additional File continues on facing page)** | | | | | | | | | | | | | | | | | | | | | | | | | | | | | | | | | | |  |
|  |  | |  | | |  |  | |  | |  | |  | |  | |  | |  | |  | | |  | |  | | |  | | |  | |  | |
| **Additional File 5. (Continued from previous page)** | | | | | | | | | | | | | | | | | |  | |  | | |  | |  | |  | | |  | | |  | |  |
| **Group** | **Gene ID** | | **SuperSage - LGE** | | |  | **RNA-Seq of lesion LCM^b^** | |  | | **Microarray - Mortel et al. [17]^c^** | | | | | | | | | |  | | | **Microarray - Schneider et al. [22]** | | | | | | | | | | | |
|  |  |  | **Incompatible reaction (PI561356-*Rpp1*)** | | |  | **PI561356 X BRS231** | |  | | **Incompatible reaction (PI230970-*Rpp2*)** | | | |  | | **Compatible reaction (Embrapa48)** | | | |  | | | **Compatible reaction (PI462312-*Rpp3* X Taiwan 80-2)** | | | | |  | | | **Incompatible reaction (PI462312-*Rpp3* X Hawaii 94-1)** | | | |
|  |  |  | **Inoculated X Mock** | | |  | **Inoculated** | |  | | **Inoculated X Mock** | | | |  | | **Inoculated X Mock** | | | |  | | | **Inoculated X Mock** | | | | |  | | | **Inoculated X Mock** | | | |
|  |  |  | **12, 24, 48h** | | |  | **10 days** | |  | | **12h** | | **120 h** | |  | | **12h** | | **120 h** | |  | | | **12h** | | **144h** | | |  | | | **12h** | | **144h** | |
| IId | Glyma09g06980 | |  | | |  | x | |  | |  | |  | |  | |  | |  | |  | | |  | |  | | |  | | |  | |  | |
| IId | Glyma05g20710 | | x | | |  |  | |  | |  | |  | |  | |  | |  | |  | | |  | |  | | |  | | |  | |  | |
| IIe | Glyma20g30290 | |  | | |  | x | |  | |  | |  | |  | |  | |  | |  | | |  | |  | | |  | | |  | |  | |
| IIe | Glyma01g43130 | |  | | |  | x | |  | |  | |  | |  | |  | |  | |  | | |  | |  | | |  | | |  | |  | |
| IIe | Glyma11g02360 | |  | | |  | x | |  | |  | |  | |  | |  | |  | |  | | |  | |  | | |  | | |  | |  | |
| IIe | Glyma16g03570 | |  | | |  |  | |  | |  | |  | |  | |  | |  | |  | | |  | |  | | |  | | | x | |  | |
| IIe | Glyma03g37870 | | x | | |  |  | |  | |  | |  | |  | |  | |  | |  | | |  | |  | | |  | | |  | |  | |
| IIe | Glyma13g36540 | |  | | |  |  | |  | |  | |  | |  | |  | |  | |  | | | x | | x | | |  | | |  | |  | |
| IIe | Glyma05g29310 | | x | | |  |  | |  | |  | |  | |  | |  | |  | |  | | |  | |  | | |  | | |  | |  | |
| III | Glyma19g44380 | |  | | |  |  | |  | |  | | x | |  | | x | | x | |  | | |  | |  | | |  | | |  | |  | |
| III | Glyma03g41750 | |  | | |  |  | |  | |  | | x | |  | |  | |  | |  | | |  | |  | | |  | | |  | |  | |
| III | Glyma04g41700 | |  | | |  | x | |  | |  | |  | |  | |  | |  | |  | | |  | |  | | |  | | |  | |  | |
| III | **Glyma08g02580** | |  | | |  |  | |  | | **x** | | **x** | |  | | **x** | | **x** | |  | | |  | | **x** | | |  | | |  | | **x** | |
| III | **Glyma05g36970** | |  | | |  |  | |  | |  | | **x** | |  | | **x** | | **x** | |  | | |  | |  | | |  | | |  | |  | |
| III | Glyma01g43420 | |  | | |  |  | |  | |  | | x | |  | | x | | x | |  | | |  | |  | | |  | | |  | |  | |
| III | Glyma16g34590 | |  | | |  | x | |  | |  | |  | |  | |  | |  | |  | | |  | |  | | |  | | |  | |  | |
| III | **Glyma09g41050** | | **x** | | |  |  | |  | | **x** | | **x** | |  | | **x** | | **x** | |  | | |  | | **x** | | |  | | |  | |  | |
| III | **Glyma18g44560** | |  | | |  |  | |  | | **x** | | **x** | |  | | **x** | | **x** | |  | | |  | |  | | |  | | |  | |  | |
| III | Glyma14g36430 | |  | | |  | x | |  | |  | |  | |  | |  | |  | |  | | |  | |  | | |  | | |  | |  | |
| III | Glyma06g14720 | | x | | |  |  | |  | | x | | x | |  | | x | |  | |  | | |  | |  | | |  | | |  | |  | |
| III | Glyma04g40130 | | x | | |  |  | |  | | x | | x | |  | | x | |  | |  | | |  | |  | | |  | | |  | |  | |
| ^a^The expression data were obtained from four global expression experiments: SuperSAGE available at www.lge.ibi.unicamp.br/soja/, RNA-Seq of microdissected lesions and two different microarrays available in the current literature. (x) indicate significant differences (p<0.05). Genes in bold were used in further analyses. Genes were ordered according to the clustering analysis. | | | | | | | | | | | | | | | | | | | | | | | | | | | | | | | | | | |  |
| ^b^LCM: laser capture microdissection. | | |  | | |  |  | |  | |  | |  | |  | |  | |  | |  | | |  | |  | | |  | | |  | |  | |
| ^c^Some probes hybridize to more than one gene. | | | | | |  |  | |  | |  | |  | |  | |  | |  | |  | | |  | |  | | |  | | |  | |  | |
